# Metallic Microneedles for Transdermal Drug Delivery: Applications, Fabrication Techniques and the Effect of Geometrical Characteristics

**DOI:** 10.3390/bioengineering10010024

**Published:** 2022-12-23

**Authors:** Nikoletta Sargioti, Tanya J. Levingstone, Eoin D. O’Cearbhaill, Helen O. McCarthy, Nicholas J. Dunne

**Affiliations:** 1 School of Mechanical and Manufacturing Engineering, Dublin City University, Collins Avenue, D09 Y074 Dublin, Ireland; 2 Centre for Medical Engineering Research, School of Mechanical and Manufacturing Engineering, Dublin City University, Stokes Building, Collins Avenue, D09 Y074 Dublin, Ireland; 3 UCD Centre for Biomedical Engineering, School of Mechanical and Materials Engineering, University College Dublin, D04 R7R0 Dublin, Ireland; 4 Advanced Manufacturing Research Centre (I-Form), School of Mechanical and Manufacturing Engineering, Dublin City University, D09 Y074 Dublin, Ireland; 5 Advanced Processing Technology Research Centre, Dublin City University, D09 Y074 Dublin, Ireland; 6 Biodesign Europe, Dublin City University, D09 Y074 Dublin, Ireland; 7 School of Pharmacy, Queen’s University Belfast, Belfast BT9 7BL, UK; 8 School of Chemical Science, Dublin City University, D09 Y074 Dublin, Ireland; 9 Trinity Centre for Biomedical Engineering, Trinity Biomedical Sciences Institute, Trinity College Dublin, D02 PN40 Dublin, Ireland; 10 Department of Mechanical and Manufacturing Engineering, School of Engineering, Trinity College Dublin, D02 PN40 Dublin, Ireland; 11 Advanced Materials and Bioengineering Research Centre (AMBER), Trinity College Dublin, D02 PN40 Dublin, Ireland

**Keywords:** micro-sized needles, solid microneedles, metallic microneedles, additive manufacturing, transdermal drug delivery

## Abstract

Current procedures for transdermal drug delivery (TDD) have associated limitations including poor administration of nucleic acid, small or large drug molecules, pain and stress for needle phobic people. A painless micro-sized device capable of delivering drugs easily and efficiently, eliminating the disadvantages of traditional systems, has yet to be developed. While polymeric-based microneedle (MN) arrays have been used successfully and clinically as TDD systems, these devices lack mechanical integrity, piercing capacity and the ability to achieve tailored drug release into the systemic circulation. Recent advances in micro/nano fabrication techniques using Additive Manufacturing (AM), also known as 3D printing, have enabled the fabrication of metallic MN arrays, which offer the potential to overcome the limitations of existing systems. This review summarizes the different types of MNs used in TDD and their mode of drug delivery. The application of MNs in the treatment of a range of diseases including diabetes and cancer is discussed. The potential role of solid metallic MNs in TDD, the various techniques used for their fabrication, and the influence of their geometrical characteristics (e.g., shape, size, base diameter, thickness, and tip sharpness) on effective TDD are explored. Finally, the potential and the future directions relating to the optimization of metallic MN arrays for TDD are highlighted.

## 1. Introduction

There are many approaches currently used for the delivery of drugs and therapeutic agents including oral administration, conventional hypodermic needles, topical creams and transdermal patches [1]. Oral drug delivery is considered one of the most desired routes of administration when compared to other routes due to high patient compliance, cost-effectiveness, less sterility constraints, flexibility in dosage delivery and the ease of production. However, it results in the poor bioavailability of drugs due to factors relating to dissolution, permeability, and solubility [2]. Conventional hypodermic needles can cause pain to the patient as they penetrate deep into the dermis where pain receptors are present. Their use is particularly challenging for needle phobic patients. The traditional use of subcutaneous injection for the delivery of macromolecules also has safety concerns for healthcare workers as needle stick injuries are a common occurrence. In some cases, subcutaneous injections are expensive as there may be a need for multiple or chronic administration by trained medical professionals [3]. The topical application and administration of drugs, using a topical cream, gel or ointment or a non-invasive transdermal patch, allows for the penetration of the drug into the skin without pain [4]. These topical methods have a limited ability to administer drugs with large particles (e.g., nucleic acids, large drug molecules) as the stratum corneum layer of the skin acts as a natural barrier [5]. The ability of a drug to penetrate the skin is influenced by the skin physiology and permeability, and various other factors including the physiochemical properties of the drug (i.e., size, molecular weight, concentration, partition coefficient and solubility) and formulation characteristics (i.e., release rate, ingredients and the presence of permeation enhancer) [6]. Additionally, the administration of ionic drugs, drugs of high concentrations or with very low/high partition coefficient can create problems such as skin irritation, non-systematic circulation and poor permeability [7]. Overall, while these topical methods have the advantage of being painless, they lack bioavailability and can lead to skin irritations, allergic reactions or non-controlled drug release [8]. 

As a result of the disadvantages of existing techniques, there is an increasing imperative for innovative methods for the delivery of therapeutic agents. For this reason, many studies have focused on the investigation of microneedle (MN) arrays as transdermal drug delivery (TDD) systems. MN arrays are minimally-invasive devices that can penetrate the stratum corneum, one of the most important barriers for topically-applied drugs, thus creating a pathway for drug permeation to the dermal tissue below [9]. MN arrays can enhance skin permeability compared to non-invasive patches enabling a faster onset of action and good bioavailability. The use of minimally invasive MN-based transdermal patches for TDD offers several important advantages over traditional drug delivery methods. These advantages include: (1) easy and controlled drug delivery; (2) the enhancement of therapeutic efficiency with fewer side effects; (3) less pain than with traditional hypodermic needles; and (4) the maintenance of a steady plasma level of the drug [10,11]. To date, MN arrays have been used in several biomedical applications including diabetes treatment [12], cancer diagnosis and therapy [13], for infections, inflammation and chronic pain treatment and the treatment and control of obesity [14], and also for other applications including the sampling of blood and interstitial fluids [15]. However, current MN-based TDD systems have associated limitations including incomplete insertion, particularly for polymeric MNs, which results in limited drug delivery efficiency and the wastage of valuable medication [16]. 

Metallic MN offer potential to overcome the challenges associated with polymeric MNs systems. However, several existing challenges limiting the translation of metallic MN arrays as a successful TDD systems remain, including: (1) current methods for metallic MN array fabrication involve a multi–step process that is not cost-effective; (2) the lack of clinical data relating to cytotoxicity of the metals used for MN fabrication; (3) limited drug loading; and (4) challenges in maintaining mechanical properties and piercing capacity. 

Recent advances in Additive Manufacturing techniques offer an innovative platform for design optimization and the cost-effective manufacture of medical-grade metallic MN arrays [17]. This review describes the various types of MN currently in development and their potential for use clinically in the treatment of diabetes, the diagnosis and treatment of cancer, management of chronic pain and treatment of obesity. The potential advantages of metallic MN are highlighted and the different methods that have been used for the development and manufacture of metallic MN arrays as potential TDD systems are discussed. In particular, the recent advances in the Additive Manufacturing of metallic MN arrays are discussed. Additionally, this review presents a synopsis of existing and in development MNs that meet the clinical requirements with optimal design and effective mechanical and geometric properties in the therapeutic drug delivery, diagnosis and treatment of damaged or diseased tissue.

## 2. Types of Microneedle Arrays

**Figure 1:**
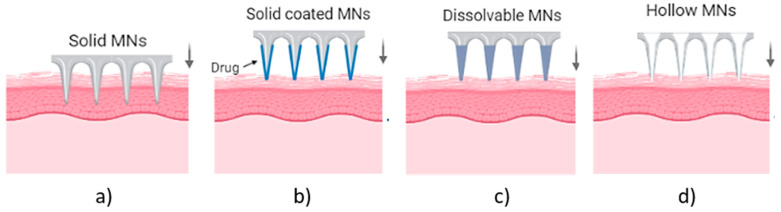
Types of MN array. (**a**) solid MNs, that require a transdermal patch for continuous drug administration after their insertion/removal, (**b**) coated, where the drug is coated around the needles, (**c**) hollow MNs for the creation of a path to administer the drug using conventional needles and (**d**) dissolvable that are filled with the drug and fully dissolved following skin insertion [14].

**Figure 2:**
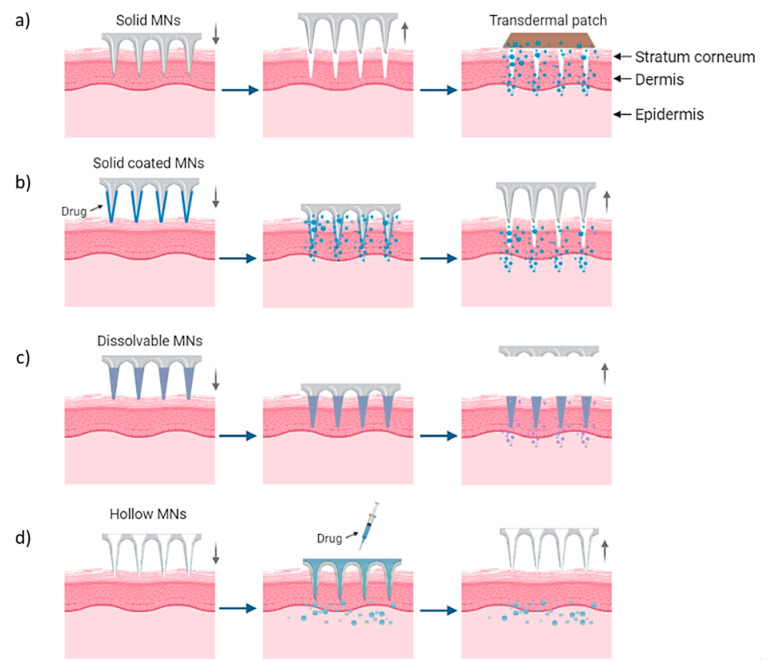
Delivery procedures of solid MNs through the (**a**) ‘poke-detach-diffuse’ method, in which the solid MNs are used only for the creation of holes and the drug administration is provided by a transdermal patch and (**b**) ‘coat and poke’ method where the drug is coated on the needles and the drug administration is provided from the MNs. (**c**) “Poke and flow” method in which the drug is inserted through hollow MNs and delivered in the body and (**d**) “poke and release” method for dissolvable MNs which are inserted and dissolved in the skin [14].

**Figure 3:**
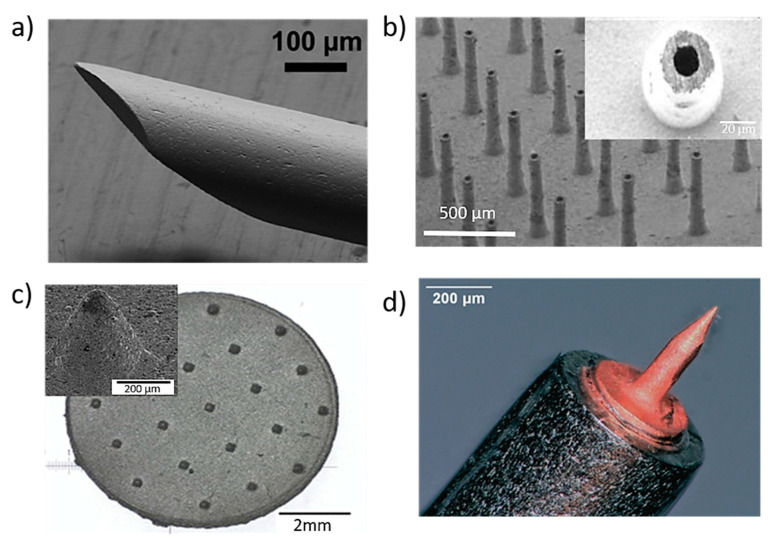
(**a**) Stainless steel MNs for blood extraction with outer diameter 210 mm and inner diameter 115 mm. Reprinted with permission from Ref. [84]. 2001, Royal Society of Chemistry. (**b**) titanium hollow microneedle array used for interstitial fluids sampling with height of 400 μm and in wall thickness 20 μm [87]; (**c**) porous 316 L stainless steel patches with 21 microneedles with a tip radius of 38 µm aiming to be used in biosensors. Reprinted with permission from Ref. [89] 2005, Elsevier Science &Technology Journals; and (**d**) cold coated additively manufactured copper needle (height = 430 µm and tip radius = 1.5 µm) aiming the inner ear application [93].

MN arrays can be characterized as: (1) solid, (2) coated, (3) hollow and (4) dissolvable (Figure 1) [18,19,20]. They can be further categorized based on their mode of drug delivery, and the materials used for their manufacture [21]. In general, there are four different modes of drug delivery: (1) ‘poke–detach–diffuse’ for solid MNs (Figure 2a); (2) ‘coat and poke’ for solid coated MNs (Figure 2b); (3) ‘poke and flow’ for hollow MNs (Figure 2c); and (4) ‘poke and release’ using dissolvable MNs (Figure 2d) [22]. The ‘poke-detach-diffuse’ method involves the use of solid MNs to create micro-channels through the epidermis into the dermis [14]. After the removal of the MN system, the drug formulation is applied to the skin surface by applying topical creams or transdermal patches and the drug is delivered through the created micro-channels [18]. Coated MN systems are solid MNs coated with a particular drug formulation. They deliver the drug during the insertion of the needles into the skin, termed the ‘coat and poke’ method [14]. The coating of the MNs can be achieved by dipping or spraying the surface of the solid MNs with the solubilized drug [23]. For coated MNs, following the penetration of the MN into the skin, the delivery of the drug is achieved by the dissolution of the coating which allows diffusion of the drug and the MNs are subsequently removed [23]. Solid MNs can be fabricated from metals (e.g. stainless steel or titanium), ceramics (silicon) and polymers (poly D, L-lactic-co-glycolic acid (PLGA) and poly-ethylene glycol (PEG)) using different fabrication methods [14,22,24]. The length and the shape of the channels formed depend on the needle geometry and design. Martiano et al. used triangular shape stainless steel MNs (height = 1 mm, width = 0.2 mm) for the TDD of insulin based on the ‘poke–detach–diffuse’ method [25]. This study demonstrated the potential for effective TDD of macromolecular drugs using solid MNs. The “coat and poke” method has also been used with titanium solid MNs of 330 µm height in an area of 1 cm^2^ coated with protein antigen for vaccine delivery [25]. The study demonstrated rapid and reproducible intracutaneous administration of dry-coated antigen [25]. 

More recently, porosity have been introduced within the structure of solid MNs, made by metals, polymers or ceramics, to enhance their ability to delivery drugs and therapeutic agents [26]. These porous MNs have different percentages of porosity, ranging between ~30% to 40% with average pore diameter 1.3 μm to 2.22 μm within their structure offering the unique ability to absorb drugs within their pores and release them upon insertion into the skin [27,28,29]. While these MNs show potential for enabling improved TDD, the volume of voids within the structure can result in MN tip collapse due to the porous structure [27]. In particular, the porosity reduced the strength to only 2 N compressive force for titanium MNs and 0.6 N for polymeric MNs. In addition to the decrease of mechanical properties, decrease of tip sharpness was also observed with increased fragility during the fabrication process [28,30]. Thus, further optimization of the selected particle size of the powder material and the pore diameter of the final part, is required to achieve porous MNs that meet the clinical requirements [27].

Hollow MN systems contain an empty cavity within the MN and a bore at the tip. Drug delivery is achieved through the ‘coat and flow’ method whereby micro-volumes of a drug can be delivered directly into the skin. They can deliver relatively large amounts of drugs with higher accuracy in dose, directly to the skin [14,18]. Hollow MNs are typically fabricated from metals and ceramics with similar fabrication techniques as used for solid MN arrays [21]. The final type of MN systems are dissolvable MNs, which are biodegradable and can be manufactured from water-soluble materials or degradable polymers [20]. The matrix of the MNs contains the drug and has sufficient mechanical stability to enable insertion into the skin, therein the matrix dissolves and the drug is released as a consequence, thus achieving drug delivery via the ‘poke and release’ method [21]. 

## 3. MN Arrays for Disease Treatment

Each of the different types of MNs previously described (i.e., solid, hollow, dissolving and coated) are fabricated from a range of different materials and have been used in drug delivery applications (e.g., insulin, growth hormones), vaccines, genes, ribonucleic acid (RNA) and proteins [14]. In biomedical applications, MNs have been used in diabetes treatment, cancer therapy and diagnosis, the treatment of chronic pain, the treatment and management of obesity and for other applications [23]. 

### 3.1. Chronic Diseases 

#### 3.1.1. Diabetes

The demand for therapies such as insulin is increasing among the adult population (9% of diabetic patients) according to the World Health Organization, with diabetes projected to be the 7th leading cause of death by 2030, with an estimated 439 million cases [31]. Diabetes mellitus is a group of metabolic diseases characterized by hyper- or hypo-glycaemia (approximately 250 million diabetic people) due to disorders in insulin secretion, action or both [32]. Currently, the treatment of diabetes involves the use of insulin delivery pumps or pens, insulin therapy, syringes, jet injectors and hypodermic needles to provide transdermal delivery of a bolus delivery of insulin [33,34]. These methods of insulin delivery pose challenges in terms of insulin administration and can lead to poor clinical outcomes [35,36]. In particular, manual techniques involve human error and patient noncompliance which can prevent optimum and accurate insulin administration from being achieved. Furthermore, injections during self-administration lead to pain [37]. A further challenge relating to insulin administration relates to the size of insulin which is a large drug molecule (i.e., molecular weight of 5808 Daltons and contains 51 amino acids) and it is difficult to deliver using transdermal delivery systems. In addition, insulin has a negative charge at pH 7.4 (i.e., anionic cargo) and as a result insulin release into the systemic circulation is slow and has poor bioavailability and cellular uptake. Limitations relating to insulin release can lead to poor glucose control in diabetic patients causing high-risk complications [12]. 

Therefore, due to the low accuracy and high risks of traditional insulin administration methods, the development of new efficient and precise insulin delivery systems is required. The development of “smart” systems capable of combining both diagnosis and therapy of Type 1 and Type 2 diabetes in response to body signals would provide a distinct advantage for patients suffering from the disease [37,38]. MNs offer the potential to deliver insulin and at the same time measure blood glucose levels, as such these “smart” systems could be an attractive solution for insulin delivery. Specifically, the MN-based arrays are capable of carrying and delivering insulin molecules, significantly reducing tissue trauma and pain [39]. 

Several studies have shown the potential of MNs to overcome the insulin administration challenges that occur using traditional methods [37,40]. In one such system, a MN array loaded with the diabetes therapy, exendin-4 (Ex4), was fabricated from hyaluronic acid using micro-molding. This system allows penetration of the skin without causing any skin damage, achieving accurate and rapid delivery [39]. Yu et al. [37] developed a glucose-responsive MN array containing glucose-responsive vesicles (GRVs) (i.e., self-assembled from hypoxia-sensitive hyaluronic acid (HS-HA)) that acts as a closed-loop system to mimic the function of pancreatic cells by combining the diagnosis and therapy of diabetes. This system provides a potentially painless way to prevent hyperglycaemia or hypoglycaemia and improve the health and life of Type 1 diabetic patients [37]. In a further study, a portable electrochemical device for Type 2 diabetes therapy with the capability to translate real time data and predict glucose levels was developed. In particular, bioresorbable polymer-based MNs, coated with the required drug, release the drug into the bloodstream when the programmed threshold temperature is exceeded. A reduction in blood glucose levels in diabetic mice was demonstrated using this device [40]. A dual mineralized MN array patch, consisting of Ex4 and glucose oxidase (GOx), enhanced the skin penetration to 100% due to the strong crosslinking of alginate-based MN arrays [41]. The study showed that painless and non-invasive transdermal administration could be achieved making it an attractive candidate for a long term, safe and on demand therapy. However, an incomplete insertion of the MN array into the skin using this system was observed. Finally, dissolvable starch and gelatin MN arrays have also been developed for the release of the encapsulated insulin, leading to complete dissolution within 5 min of insertion into the skin. This research confirmed the stability of the encapsulated insulin and great potential for its TDD in a relatively painless, rapid and convenient manner [42].

One drawback of the methods previously described is the limited ability of the MN arrays to fully penetrate the skin without plastic deformation on the tip. Therefore, research efforts have been focused on enhancing the penetration properties and the tip sharpness or failure forces of the MNs in order to improve insulin delivery. For instance, Invernale et al. fabricated platinum-coated 316 L stainless steel 2D MN arrays with a length of 680 μm and width of 250 μm for continuous glucose monitoring [43]. The MNs penetrated the skin without fracture of the needle and achieved continuous monitoring, and the reduction of glucose levels for up to 7 days. Although cytotoxicity and biocompatibility are among the greatest issues facing such MN-based sensors, the proposed technology demonstrated no significant negative impact on cell viability. Finite element models have also been applied to further investigate the performance of the metallic 2D MN arrays. In a further study, a titanium hollow patch, consisting of needles with a length of 500 μm and a tip diameter of 75 μm, achieved pain-free administration and continuous drug delivery in a diabetic rat model [44]. The MNs showed sufficient strength to pierce the skin without breaking, improving the glucose levels by 50% within 4 h with a total of 0.43 ng/mL of insulin being released. An in vivo study, conducted by Martanto et al. in a diabetic rat model, investigated transdermal insulin delivery using laser-cut solid stainless steel MNs [45]. The MNs demonstrated full penetration into the rat skin with high skin permeability to insulin. The decrease of blood glucose achieved was similar to that from subcutaneous insulin injection (e.g., 0.05–0.5 U) with 0.5–7.4 ng/mL insulin concentration. High insulin concentration with shorter insertion time was obtained within increased transdermal insulin delivery and lower blood glucose levels by as much as 80%. Porous titanium MNs have also shown promise for insulin delivery [46]. The force-displacement graph during the penetration in the pigskin (with a thickness of 1–2 mm) indicated that full penetration was achieved with a force of only ~3 N required without the fracture of the tip. Successful and efficient insulin delivery was achieved in a diabetic rat model within 2 h, decreasing the blood glucose levels to normal values.

The MNs described here have shown an ability to provide continuous drug delivery without causing any pain compared to conventional hypodermic needles. Polymeric-based MNs have difficulty penetrating the stratum corneum and they have a high risk of needle breakage [42]. Mineralized MNs, formed by the chemical crosslinking of the polymer, have limitations relating to the toxic traces from the crosslinking process, which can potentially lead to skin irritation or infections [47]. However, the limitations related to the penetration of the stratum corneum barrier and the amount of drug encapsulation that can be achieved has shown an improvement with the use of metallic MNs [48]. Overall, despite the extensive research in the field of MN technology for the treatment of diabetes, further research is required to enhance their functionality and the control of release behavior.

#### 3.1.2. Obesity

According to the World Health Organization in 2016, more than 1.9 billion adults aged 18 years and older were overweight [49]. Of these, over 650 million adults were obese. The number of incidents increased in 2019, with an estimated 38.2 million children under the age of 5 years being overweight or obese [49]. Obesity is highly connected with several diseases, namely leading to a high risk of diabetes and cancer, which are responsible for 20% of deaths in adults [50]. Different factors influence the development of obesity, such as environmental and genetic factors, as well as an imbalance between calorie consumption and energy expenditure [51]. 

Current approaches towards long-term weight management for patients suffering from obesity include diet, physical exercise, pharmacological therapy and surgical approaches. However, these current treatments are associated with low effectiveness or undesired systemic side effects. Many studies have been focused on the use of surgical treatment approaches, which involve invasive procedures and have high risks. For instance, the modification of the anatomy of the gastrointestinal tract can lead to a decrease in food absorption and consumption [52]. Inflammation, infection, serious morbidity or even death can also result from using surgical approaches. Other methods involve the use of pharmacological agents, which have the benefit of not requiring invasive procedures. However, currently, these pharmaceutical approaches include limitations, leading to inefficient results, undesired side effects on human organs such as gastro-intestine, liver and kidney and limited applications [53].

There is a clear need for new approaches that can effectively treat obesity while overcoming the significant risks arising from the traditional treatment approaches. MN-based arrays have potential as minimally invasive devices that could provide an effective approach for the treatment and control of obesity. The MN-based arrays could provide a localized, convenient, and painless administration method for pharmacological agents for the treatment of obesity. This approach would also allow the side effects relating to current surgical procedures to be avoided. A recent study reported the development of hyaluronic acid-based dissolving MN arrays for the TDD of caffeine for the treatment of obesity [54]. The results indicated the significant improvement of lipolysis resulting in a considerable weight loss leading to the 12.8 ± 0.75% reduction of obesity using the caffeine loaded dissolving MNs three times per week in obese C57BL/6J mice [54]. A further study reported the use of dissolvable MN-based arrays encapsulating rosiglitazone (Rosi) nanoparticles for the delivery of browning reagents, for the long term management of weight [55]. The browning reagents are capable of turning energy from food into heat in the body in a safe and effective manner for the inhibition of adipocyte hypertrophy improving the metabolism action. The delivery of browning agents reduced the inguinal fat pad of the obese mice during a four-week treatment achieving approx. In all, 100% normalization of the body weight and glucose levels of ~110 mg/dL from 140 mg/dL [55]. In addition to obesity treatment, this TDD approach led to improved insulin sensitivity having a positive effect not only in the fight against obesity but also against diabetes. Hyaluronic acid-based dissolvable MN patches (10 × 10 arrays) with a height of 600 μm have been used to deliver the anti-obesity compounds (i.e., β3-adrenoceptor agonist and the thyroid hormone, triiodothyronine) transdermally to white adipose tissue (WAT) to increase energy expenditure and transform the calorie-storing white fat into calorie-burning brown fat [56]. The optimal MNs required a lower effective dosage compared to systemic administration with only 5 mg at day 1, resulting in a reduction of the side effects associated with the over-activation of β3-adrenoceptors, such as increased heart rate and blood pressure. These dissolving MNs effectively promoted WAT browning and suppressed body fat and weight gain in a diet-induced obese mouse model, without the need for daily administration since a slow release of β3-adrenoceptor agonist can be achieved [56].

In a further study, An et al. developed dissolvable MNs composed of the natural polymer gelatin for the treatment of obesity [57]. The gelatin-based MNs showed a reduction of local subcutaneous fat by up to 60% compared to non-treated control samples, demonstrating the direct effects of gelatin on the fat accumulation at the applied body part [57]. This was considered as an effective and simple method to target the local reduction of subcutaneous adipose tissue.

### 3.2. Cancer Diagnosis and Treatment

Cancer is considered a major public health problem. According to GLOBOCAN data, 18 million of the world population were diagnosed with cancer prior to 2018, with 0.5 million new cases in 2018 [58]. While cancer vaccination has been demonstrating promising anticancer results, it is predicted that by 2040 cancer incidence will have increased by 11.4 million new cases worldwide in total [23]. Traditional cancer treatment includes surgery, chemotherapy and radiotherapy approaches, which often lead to side effects (e.g. drowsiness, exhaustion, vomiting, etc.), acute toxicity or tumor recurrence [13]. An appealing solution would be the use of a controllable and easily applicable minimal invasive process (i.e., MN-based arrays) for immunotherapy and gene-based therapy for both cancer vaccination and treatment [13]. 

The treatment of cancer using MN arrays has garnered significant attention by the scientific community, resulting in many studies being conducted on the application of MN patches containing immunostimulatory adjuvants and/or antigens as anticancer therapeutic approaches capable of achieving the clinical requirements (Table 1). For instance, drug-loaded dissolving MN patches comprised of hyaluronic acid have been developed for the treatment of multiple tumors and the delivery of long-term treatments [59]. These MNs were shown to be light-activated without affecting the structure of the MN array, or their ability to penetrate the skin, able to achieve highly controllable on-demand delivery and to dissolve after the release of cargoes within the tumor site. These dissolvable MN arrays present a promising platform for the treatment of superficial skin tumors providing safety, effectiveness, good handling properties, low-toxicity and a minimally invasive approach [60]. Ye et al. reported the use of dissolvable hyaluronic acid-based MN arrays for the delivery of immunotherapy for the treatment of melanoma [61]. Maaden et al. used fused silica hollow MNs for the delivery of a therapeutic cancer vaccine, achieving stronger functional cytotoxic and T-helper responses in mice, while requiring lower volumes compared to traditional intradermal immunization [62]. 

Chablani et al. developed metallic MNs, 1100 μm in length, for the treatment of breast cancer [63,64]. These metallic patches demonstrated the successful delivery of a particulate breast cancer vaccine through 50 μm holes created in the skin. In vivo assessment using a mouse model showed five times more tumor suppression in vaccinated animals, confirming successful immune response activation and protection. The same metallic MN array system were used for the transdermal delivery of an ovarian cancer vaccine by Tawde et al., achieving high tumor suppression [65]. The use of coated metallic MNs for the improved delivery of a skin cancer drug has also been reported [66]. These MN arrays significantly suppressed tumor growth within 5 min with a delivery efficiency of 90%. Gill et al. fabricated and investigated coated stainless steel MNs for the delivery of different proteins (e.g., calcein, vitamin B etc.) and DNA. The coating was absorbed within 20 sec, achieving a controllable and rapid delivery into the skin [67].

Overall, these studies indicate that the use of MN array systems for immunotherapy, gene-based therapy and vaccine administration can be considered as a highly attractive approach, which requires further investigation.

### 3.3. Chronic Pain

Pain, acute and chronic, is the main reason for daily suffering for approximately 1.5 billion people, regardless of age [13]. Sources of pain are commonly the result of trauma, infections, inflammation, tumors, metabolic disease or endocrine diseases [65]. Acute pain is usually associated with an injury and has a short duration, whereas pain that persists for a longer duration, e.g., greater than three months is considered chronic. The conventional method for the delivery of analgesics for the temporary relief of acute and chronic pain is through oral administration or/and injection. The use of MN patches provides a promising technology for pain management offering additional benefits over the conventional methods, including a reduced risk of systemic side effects. According to Xie et al. [68] the fabrication of dissolvable MNs made of sodium carboxylmethyl cellulose (SCMC, molecular weight ~90,000) has been shown to be safe and effective for the local delivery of anti-calcitonin gene-related peptide (CGRP) that produces selective anti-hypersensitivity through antagonism of peripheral CGRP receptors for the treatment of neuropathic pain. In these studies, the use of the MNs resulted in significant pain relief without skin irritations in contrast with traditional treatments, while avoiding the side effects associated with systemic exposure [69]. Lidocaine works as an analgesic for both chronic and acute pain, which is administrable via injection or non-invasive transdermal patch. The topical delivery of lidocaine using non-invasive transdermal patches is less efficient than traditional injections due to the low drug permeation (i.e., the undesired delay of drug release) and the variability of drug absorption among individuals. Hence, to address the clinical problem, a solid drug-loaded integrated transdermal polymer-based MN patch was developed. Briefly, poly(ethylene glycol) diacrylate, (PEGDA (M_n_ = 258)), 2-hydroxy-2-methyl-propiophenone (HMP) were mixed and exposed to a high power ultraviolet (UV) light source leading to a significantly higher amount of drug encapsulation and a faster onset of drug permeation compared to commercial patches. These MNs demonstrated an appropriate release and delivery of the active ingredient (lidocaine) for the relief of acute and chronic pain [70]. The development and use of a photo-triggered dissolvable polycaprolactone (PCL)/poly(l-lactide-co-d,l-lactide) (PLA) MN system for the on-demand lidocaine delivery has also been reported [71]. Specifically, more convenient, comfortable and effective pain control than standard injection was achieved with the use of this system, with rapid absorption into the blood circulation and high bioavailability (95%) reported. 

**Table 1:** Microneedles used in vivo as vaccines for cancer therapy.

Materials	No. of MNs	Therapy Agent	Height–Width	Results
DEXTRAN/polyvinyl-pyrrolidone, and hyaluronic acid	2 × 12	STAT3 siRNA/polyethylenimine complexes	h: 650 µm w: 300 µm	Reduction of tumor growth, tumor volume and weight, by ~80% with total dose of 264 µg of STAT3 siRNA and by ~50% total dose of 132 µg of STAT3 siRNA [72]
Polyvinyl alcohol	19 × 19	RALA/E6 and E7 pDNA	h: 600 µm w: 300 µm	The use of MNs decreased the tumor weight (i.e., 3.6 fold smaller) compared to control mice [73]
Pluronic F127/Poly (ethylene glycol)	7 × 7	OVA and R848	h: 350 µm	Administration of OVA/R848 using the MN patch, resulted in a significant delay of tumour growth (tumor size: ~500 mm^3^ after 25 days) compared to control mice (tumor size: ~3000 mm^3^ after 25 days) [74]
Polyvinyl pyrrolidone	19 × 19	RALA-E6/E7 DNA nanoparticles	h: 600 µm w:300 µm	Increase the percentage of survival by 40% after 40 days with the use of nanoparticle-MNs [75]
Hyaluronic acid	9 × 9	aPD1, glucose oxidase, anti-CTLA4 antibody	h: 600 µm w: 300 µm	Treatment with aPD1-GOx-MN patch show that 50% of mice survived with undetectable tumor after 40 days. Complete control of melanoma & disease-free survival of approx. 70% of mice in 60 days with the use of aCTLA4 and aPD1 MNs [76]
Hyaluronic acid	15 × 15	1-methyl-DL tryptophan and aPD1	h: 800 µm w: 300 µm	Reduced tumor growth (tumor area: less than 100 mm^2^) compared to the control (tumor area: ~300 mm^2^). While at the same time 40 days after the treatment 70% of mice survival was observed [61]
Methylvinylether and maleic anhydride	19 × 19	Ovalbumin loaded poly(D,L-lactide-co-glycolide) nanoparticles	h: 600 µm	Delay of tumor growth (tumor volume: 10 mm^3^) during the 13 days of treatment [77].
Poly(D,L-lactide-co-glycolide), poly(β-aminoester), poly(4-styrene sulfonate) and protamine sulphate	19 × 19	pDNA and poly(D,L-lactide-coglycolide) nanoparticles	h: 650 µm w: 250 µm	Complete loss of pDNA coating from the surface of the MNs and transferred in the epidermis after 24 h [78]
AdminPen	43	Microparticle loaded with whole cell lysate of ID8 ovarian cancer cells	h: 1100 nm	Decreased tumor growth with transdermal vaccination (tumor volume: ~300 mm^3^) compared to placebo vaccination (tumor volume: ~500 mm^3^) after 15 days [65]
AdminPatch-1200	43	Microparticle loaded with drug proteins or DNA	h: 1100 nm	Five times more tumor suppression than the control animals confirming the immune response activation and protection [64].

### 3.4. Other Applications

MNs have also been extensively studied for a number of other biomedical applications, including blood and interstitial fluid sampling and for biosensing applications. MNs can be used for blood sampling since the blood capillaries are presented under the dermis at a depth of 400 µm. However, the tips of the nerves are in a similar depth and thus some of the MNs in the array might graze the uppermost nerve cells [12]. A study exploring the effect of MN length on pain reported that needles ranging from 480 µm to 1450 µm resulted in pain scores of 5% to 40% when using a 26-gauge hypodermic needle [79] and thus minimizing the MN length is important to result in a minimum of pain. The use of MNs for blood sampling resulted in a reduction of pain by up to eight times when compared to conventional hypodermic needles [80]. In order to extract blood, MNs require mechanical and penetration properties that can achieve skin penetration without buckling. For example, an average force value for human skin puncture is 6.0 N, it is 2.0 N for subcutaneous fat tissue and 4.4 N for muscles and therefore metallic MNs are usually used [81]. These MNs require a large inner diameter (40 µm–125 µm) to allow the largest blood cells to pass while at the same time preventing the risk of failure due to buckling [82]. Many factors can affect the process of the blood extraction, such us the fluid density, the MN diameter and the material. The use of a microfluidic pumping device can improve the process as well as the capillary action, a natural action, which is enough for the blood extraction process [12]. Kawanaka et al. developed titanium MNs (inner Ø 100 μm) with a vacuum chamber for blood extraction [83]. The results show a good volumetric efficiency up to 90%. Another study presented stainless steel MNs (outer Ø 210–250 μm) as a blood/interstitial fluid extraction system for pharmacokinetic studies (Figure 3a) [84]. The in vivo studies on mice presented the ability of the MNs (inner Ø 115 µm) to collect successfully the blood through capillary action within a 1 µL steel reservoir.

Similarly, for the sampling of interstitial fluids, the MNs have to penetrate the stratum corneum and reach the dermis layer where the blood vessels are [85]. The sampling can be achieved through capillary action combined with a pumping device, however this method is not cost-effective due to the price of the device [12]. While, for the MNs, the capillary forces enhance the hydrophilicity and help with the extraction of the fluids leading to faster and easier blood extraction without the use of any device that makes it cost effective [84]. Mishra et al. tested SU-8 microneedles with 500 μm height and 40 μm inner diameter for the optimization of the hollow microneedles design [86]. The flow rate was tested resulting in 0.93 μL/s through the microneedle lumen [86]. In contrast, Kim et al. developed hollow metallic 10 × 10 MN arrays with a height of 400 µm and smaller inner diameter of 20 µm for the extraction of interstitial fluids (Figure 3b) [87]. These type of MN arrays delivered the fluids in a controlled manner with a fluid flow rate of 0.69 µL/s. For interstitial fluid extraction, the inner diameter of the MNs plays a significant role, whereby decreasing the diameter led to a decrease of the flow rate through the MN.

In addition, MNs have been used as biosensors providing the ability for minimally invasive measurements of biological or chemical reactions and monitoring. Metallic-based solid, hollow and solid porous MNs made by stainless steel, platinum, carbon and gold have been fabricated as biosensors [88]. Biosensors are assembled using hollow MN by placing wires of platinum, silver or carbons inside the channel of hollow needles. On the other hand, solid MNs have been molded using the necessary biological recognition elements [88]. Cahill et al. fabricated porous 316 L stainless steel MNs designed for the storage, delivery and absorption of fluids inside the porous network for biosensing (Figure 3c) [89]. These MN arrays were fabricated using hot embossing of the patch followed by sintering at 1100 °C and electropolishing at 2 A and 10 V for 1 min. They achieved porosity up to 36% with average pore size Ø 2.22 µm and 27 ± 5 µL of fluid was able to wick up through capillary action.

Another challenging application is the accessibility of the inner ear for the delivery of drugs due to the fluids that exist in the cavity within the temporal bone. The only way to reach the inner ear without the breakage of bone is the round window membrane (RWM) in the middle ear. Studies have explored the use of polymeric MNs, with tip Ø of 500 nm, for the delivery of drugs for this challenging application [90]. Due to the risk of the tip/needle failure because of the lack of mechanical strength (~0.2 N/needle, typical failure force under axial load [91,92]) of the polymeric materials, further research has led to the development of gold-coated copper-based MNs for the penetration of the RWM (Figure 3d) [93]. In this study, MNs with a height of 430 μm and tip radius of 1.5 µm were fabricated using an electrodeposition process. The ultra-sharp MNs were tested in guinea pigs and achieved a mean perforation force (i.e., maximum force that is exerted on the needles during the indentation) of 3.8 ± 0.3 mN. While a minimal degree of trauma was observed since the shaft diameter was 100 μm without introducing uncontrolled ripping of the RWM and at the same time, the MNs remained undamaged. 

## 4. Metallic Microneedles

Although MN-based arrays fabricated from a range of materials have shown promise for clinical application in the treatment of a range of diseases, they still lack of mechanical properties. Particularly polymeric MNs, presents challenges in terms of repeatable penetration, with incomplete insertion affecting the volume of drug loading that can be achieved, resulting in inconsistent drug delivery [23]. The use of citric acid as a crosslinking method to improve the mechanical properties can result in biocompatibility issues due to toxic traces from the crosslinking process remaining on the MN surfaces [94]. 

Metallic MNs offer a number of advantages of polymeric MNs. While high start-up costs are initially required for the fabrication of metallic MNs and post-processing following fabrication is normally necessary, they have shown considerable promise as efficient minimal invasive delivery systems [30]. Metallic MNs provide high mechanical strength, are biocompatible and offer potential for drug elution [66]. Due to their high mechanical strength, they can easily penetrate the stratum corneum without failing and can effectively create pathways across skin enhancing drug flux [66]. Metallic MNs have higher fracture toughness and yield strength values (greater than 200 MPa) than polymeric MNs. They have been shown to exhibit a high degree of dimensional accuracy and reproducibility, while their unique physical and chemical properties afford them the ability to deliver small, lipophilic molecules through soft tissues such as skin to a range of organs [18,24]. 

A number of manufacturing techniques have been employed including stereolithography, laser cutting and ablation, wet/dry etching, metal electroplating, injection molding and laser sintering, leading to metallic solid or hollow MNs with complex geometries [25]. 

### Metals Used for Microneedles

A range of metals including stainless steel, titanium, palladium, nickel, tantalum, platinum and palladium–cobalt alloys have been successfully used for biomedical applications [21,95]. For the manufacture of MN arrays, stainless steel and titanium are most commonly used in research at the present time. Compared to stainless steel, titanium can lead to lower corrosion rates combined with higher mechanical properties resulting in the development of solid applications, while hollow applications can be also used as base of the coated MNs [95]. In addition to their good mechanical properties and biocompatibility, stainless steel and titanium offer high strength, allowing penetration of the skin without fracture of the MN-based arrays, thus offering advantages compared to counterparts composed of other materials [96]. Nickel has issues relating to biocompatibility, leading to the risk of toxicity so requires careful use [45]. For instance, nickel sensitivity or development of dermatitis have been observed following contact with alloys containing approximately 14% nickel [97,98]. Meanwhile, even MNs containing a low percentage of nickel (8%) have been reported to cause allergic contact dermatitis [99]. Palladium and platinum have not been adequately studied for use in the manufacture of MNs [100]. A more detailed comparison of the mechanical properties of the metals used for MN manufacture is presented in Table 2.

**Figure 4:**
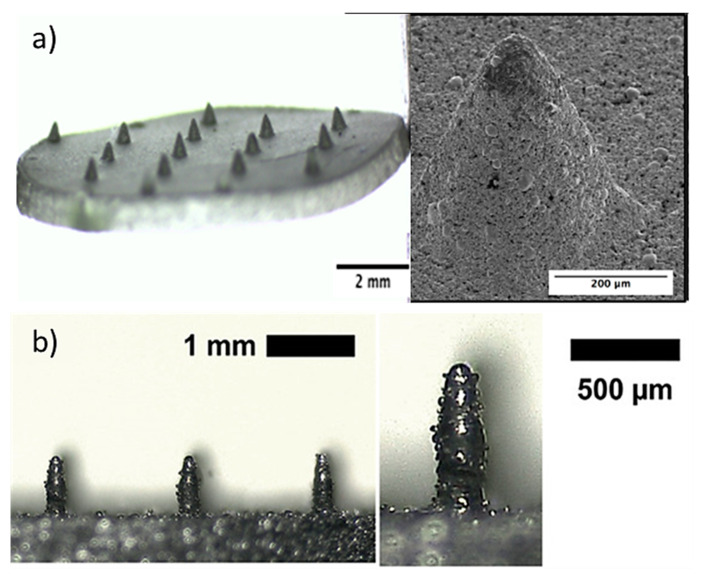
Stainless steel (**a**) porous MNs used for storage, delivery of drugs and absorption of fluids for biosensing produced by the hot embossing technique. Reprinted with permission from Ref. [27]. 2005, Elsevier Science &Technology Journals, (**b**) solid conical–shaped MN electrodes in a rectangular patch produced by direct metal laser sintering (DMLS) 3D printing process. Reprinted with permission from Ref. [104]. 2020, Advanced Materials Technologies.

Stainless steel is an iron-based alloy that contains a high percentage of chromium (11–30 wt. %) and varying amounts of nickel [21]. For this reason, the composition of stainless steel affects its biocompatibility. The optimal biocompatibility is provided by 316 L stainless steel, which is also widely used for endovascular devices and surgical applications. Nevertheless, this is not critical for MN arrays since their application does not require long-term contact time with the body. Their use is similar to traditional stainless steel (304 SS) hypodermic needles, which are commonly used and considered as biocompatible [96,101]. Due to the high biocompatibility and mechanical properties (e.g., Young’s modulus of 200 GPa) of stainless steel, it was the first metal used in the manufacture of MN arrays [102,103]. The simplest method for the fabrication of stainless steel MN arrays involves holding traditional hypodermic needles in a supporting material and changing the length of needles [96]. More advanced stainless steel MNs with a variety of designs and geometries (Figure 4) can be produced using microfabrication technology [103].

An alternative to stainless steel-based MN arrays, are those produced using titanium. Titanium and its alloys (e.g., Ti-6Al-4V ELI) are used in applications such as hip prostheses and pacemaker cases [105] and have a reduced Young’s modulus and percentage of elongation (102–120 GPa), (10–30%), respectively, compared to stainless steel (193–200 GPa), (60–70%) [102], while offering sufficient strength to avoid breakage during the implantation process. Therefore, titanium and its alloys are suitable materials for the fabrication of MN arrays. As a result, a range of titanium-based MNs are being developed as TDD systems [106] and as biosensors [21]. Both titanium alloys and stainless steel offer high levels of corrosion resistance due to the formation of a surface oxide layer, which makes them safe for use in humans and animals [21]. Although there are many studies investigating the clinical performance of titanium and titanium alloys more studies and work for orthopedic and dental applications is required [107,108]. In addition, there are limited clinical studies relating to the application of similar materials for the application of TDD using MNs fabricated from titanium and titanium alloys. 

## 5. Fabrication Methods for Metallic MNs

Technological developments in the microelectronics industry between 1990 and 1999 have led to new microfabrication tools suitable for producing MNs for medical applications [101]. The first MNs produced were manufactured from silicon and since then different types of materials, such as metals, polymers, ceramics and glass, have been used for MN fabrication. The fabrication of metallic MNs is focused on achieving the optimal MN geometry, shape, size and tip sharpness to increase the mechanical strength and reduce the required force for the MNs penetration into the skin [102]. Methods used for the fabrication of MNs include AM techniques (3D printing), such as direct metal laser sintering (DMLS), laser cutting, laser ablation, etching, electroplating, hot embossing and metal injection molding (MIM). These fabrication techniques are summarized in Table 3. 3D printing offers particular advantages such as manufacturing versatility and customisability, as well as the ability to manufacture complex structures to a high accuracy and precision (e.g., dimensional fidelity within ±5% of desired dimensions). Furthermore, the fabrication of MNs using 3D printing enables the rapid modification of key properties that influence the MN performance. To date, there are a number of different techniques that are used for the fabrication of metallic MNs include laser cutting, laser ablation, etching, electroplating, hot embosing and injection molding. All the different fabrication techniques are summarized in Table 3. 

### 5.1. Direct Metal Laser Sintering (DMLS) 

**Figure 5:**
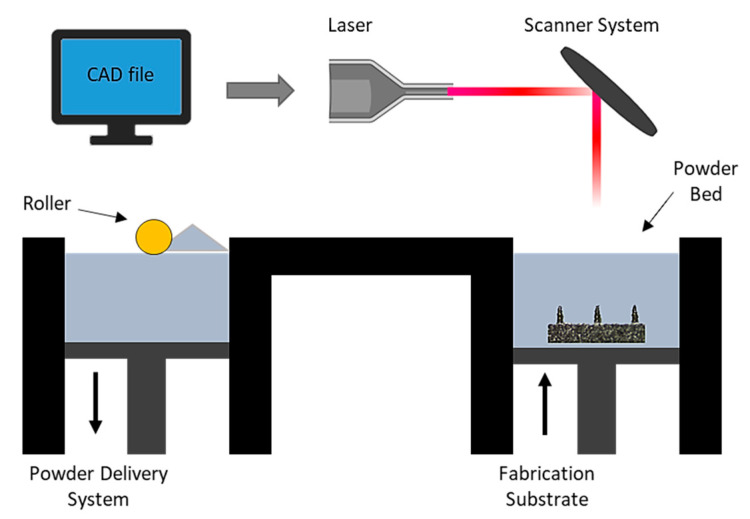
Schematic illustration of the DMLS process which is a layer by layer sintering process using a computer control to import the CAD file, while a laser beam directly melting the first layer of metal powder. A powder delivery system and a roller are used to apply the next layer of metal powder until the final part is produced [113].

**Figure 6:**
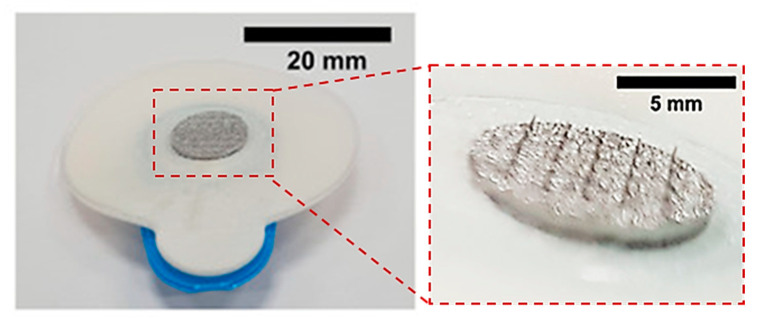
3D printed 5 × 5 MN electrodes used for measurement of electrical muscle activity in humans. Reprinted with permission from Ref. [104]. 2020, Advanced Materials Technologies.

Direct metal laser sintering (DMLS) is an AM technique for the fabrication of metallic parts. DMLS is a power bed fusion technology that can combine this single fabrication step with the ability to produce three-dimensional parts [109]. It is one of the most widely used technique due to the accuracy in the geometry, low cost and the ability to provide parts close to the shape and aspect ratio of the final product (near net shaped parts) [110]. This technique has already been used in medical applications, particularly for orthopedic and dental applications [111,112]. The process uses a laser beam in order to melt together different layers of powder to a required layer thickness as specified in the computer-aided design data (Figure 5) [110]. Krieger et al. reported the fabrication of 316 L stainless steel MNs using DMLS technology followed by electropolishing [104]. In this study, they developed 3D-printed dry MN electrodes, 5 × 5 MN arrays, using a laser power of 55 W, for the measurement of electrical muscle activity in humans (Figure 6). This study presented high quality surface electromyography signals, with reduced electrode–skin contact resistance at approximately 63%, showing the potential of these MN electrodes to be used for the application of biosignal recording.

### 5.2. Laser Cutting

Laser cutting is applied in different manufacturing processes due to its high cut quality [103]. This process is cost-effective due to the large amounts that can be produced in industry. This method is widely used for the manufacturing of solid metallic MN arrays. Firstly, the appropriate geometry and dimensions of the needles are designed in a computer aided drawing (CAD) software (e.g., SolidWorks, AutoCAD, etc.). Stainless steel MN arrays are usually produced by laser cutting while a laser beam melts the material and an assist gas (i.e., oxygen, nitrogen, etc.) blows the melt in the shape of the needle into a plane sheet and then bends them, usually at a 90° angle, out of the plane of the sheet [105]. However, this procedure leads to poor surface finishing of the MN arrays. To decrease the surface roughness from deposited oxides, the MN arrays have to be cleaned with detergent followed by rinsing in running water [106]. Post-processing (i.e., electropolishing) is required for the cleaning of the edges of the MN arrays as well as for sharpening of the MN tips.

**Figure 7:**
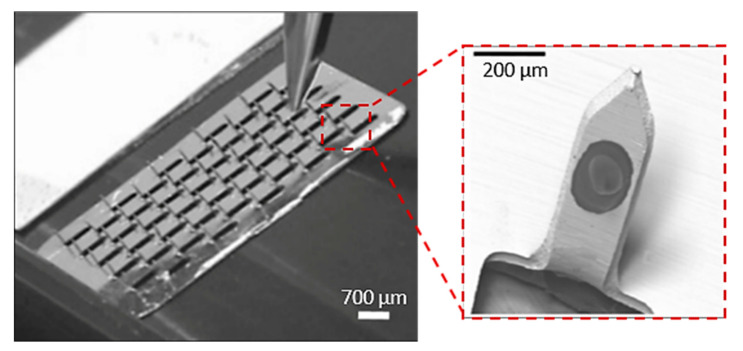
MNs arrays, used as drug carrier of anticancer drug formulations, (50 needles in total) produced by laser cutting method by cutting the shape of the needles in the stainless steel sheet and bent the needles out of plane at 90°. Reprinted with permission from Ref. [114]. 2015, Elsevier.

MN arrays with different dimensions have been successfully produced with the use of the laser cutting process. Uddin et al. built micro-sized needles with 700 µm length, 200 µm width and 50 mm thickness from stainless steel sheets (SS 304) by the laser cutting process (Figure 7) [114]. Initially, a CAD model was desined with the desirable needle geometry, and then the laser beam traced the shape of the needles on the metal sheet creating the MNs. In order to create the final MN arrays with 50 MNs in total, the needles were manually bent at 90° out of the sheet plane. The MN arrays were then drug coated for the examination of drug release via the “coat and poke” method through murine skin [115]. Rapid release rates were obtained with approximately 85–90% of the drug being released after 1 h and 100% of the drug released within 3 h. It was also observed that application marks, and drug residue on the skin, disappeared after 24 h. 

### 5.3. Laser Ablation 

**Figure 8:**
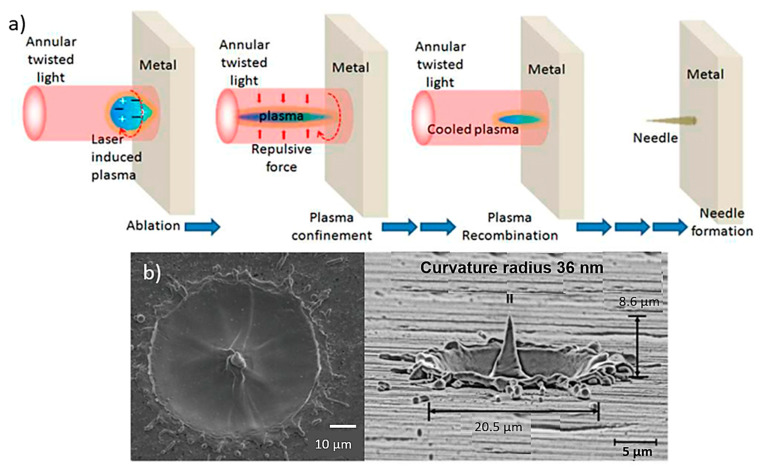
(**a**) Schematic for MN fabrication using light pulses that are able to give the suitable needle shape in the sheet [118], (**b**) produced metallic needles by the laser ablation technique with max height of 8.6 μm using three light pulses [119].

Laser ablation techniques are usually used for thin metal films (e.g., stainless steel and tantalum) where the excimer laser ablation is able to isolate adjacent MN arrays [30,114]. Laser ablation can be used to fabriacte clean and precise structures due to direct solid-to-vapour transformation in low pulse energy and heat [116]. In this process, the laser is focused on the metallic sheet and after a single-shot, a protuberance appears at the centre of the metallic sheet (Figure 8a) [100]. The light pulses are able to give the suitable shape in the sheet and after three pulses the protuberance takes the shape of a MN with a 10 µm height approximately. The height of the needles changes with the deposition of more or less light pulse [117].

MN fabrication on a metal surface based on laser ablation using twisted light with spin was demonstrated, for the first time by Bhattacharya et al. [117]. The resulting needle showed a height of at least 10 µm above the target surface and a tip diameter of less than 0.3 µm. The needles were uniformly well shaped with an average length and tip diameter of approx. 10 µm and 0.5 µm (Figure 8b).

### 5.4. Etching 

Etching techniques are used for the fabrication of titanium-based MN arrays but also for the sharpening of the tips of already fabricated MNs [19]. There are two types of the etching process, i.e., (1) wet and (2) dry etching. The material that is used for the fabrication of the MNs defines the selection of the process [120]. For the wet etching process, liquid chemicals or etchants are used to remove the material from a wafer [21]. During the process of wet etching, the specific shape (e.g., of the MN) is defined by photoresist masks on the wafer. The liquid chemicals etch the pattern that is not covered with the photoresist masks. In this way, the liquid chemical reacts with the material and then the oxidized material is dissolved. At this stage, the photoresist mask will become detached leading to the form of the final product (i.e., MN arrays) [118]. The dry-etching process involves usually a plasma of reactive gases (e.g., fluorocarbons, oxygen, chlorine, nitrogen and argon), while the ions, that the material is exposed to, are able to etch the surface and remove the material in a specific pattern giving the final shape of a MN [120].

**Figure 9:**
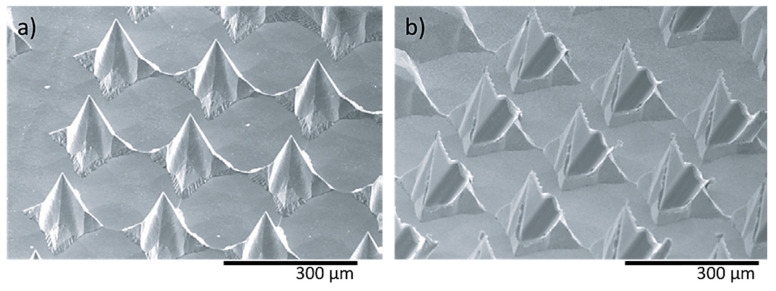
Nickel MN arrays (**a**) pyramid shaped needles (needle height: 120 μm) and (**b**) flattened needles (needle height: 170 μm), fabricated by the etching process for supplying medical solutions. Reprinted with permission from Ref. [122]. 2006, Journal of Micromechanics and Microengineering.

In a recent study, two different shaped needle structures, i.e., pyramidal (Figure 9a) and flattened (Figure 9b) needles, were fabricated using wet etching for the determination of the mechanical characteristics [121]. The height and pitch of both needle types were 120–250 μm and 170–280 μm, respectively. The compression strength achieved was approximately 40 mN for individual MNs and 10 N for a total array of 160 needles.

### 5.5. Electroplating

The electroplating process, also known as electrodeposition, is suitable for the fabrication of both solid and hollow MNs (e.g., palladium and copper) [19,100]. This process is based on a liquid solution of ionic species, in which a charge transfer during the deposition occurs to produce the metal or oxide layer in the electrode [123]. An electrolytic bath solution with positively charged ions is used, in which the positively charged ions are reduced from an applied external electric field in a way that the metallic material is electrodeposited to individual solid or hollow MNs [124]. The thickness and the deposition rate of the material can be controlled with the change of the electrodeposition time. The increase of the time of the reaction leads to the consumption of more material resulting in increased final product thickness [122,123].

**Figure 10:**
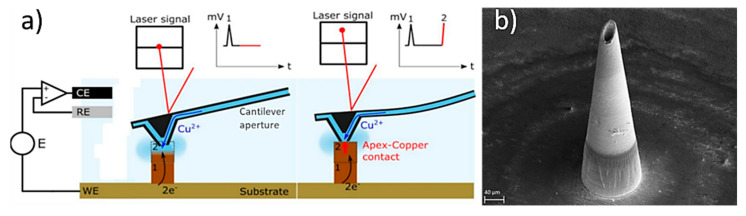
(**a**) Schematic of the printing process inside the printing chamber, whereby the copper ions are injected through a cantilever aperture to create the final part. Initially the computer provide the order to the positioning control which regulates the additive manufacturing process by an embedded controller, the cantilever is moving using the Z-stage inside the printing chamber, and the electrolyte flow via the cantilever is modulated by the microfluidics control, and (**b**) hollow MN produced by the electroplating/Additive Manufacturing process [125].

In a study, Sachan et al., manufactured copper hollow MNs with an Additive Manufacturing approach based on the electroplating process [125]. This method combines fluid scanning probes with the 3D printing process. During the process, copper ions are injected through a cantilever aperture inside of the printing chamber of the 3D printer (e.g., CERES system), and the ions impound the electrochemical copper reduction into a small region creating a unit building block (voxel) (Figure 10a). When this reduction reaches the cantilever, which is located within the printing head, it is deflected and starts positioning to the next location. Once a voxel is completed the tip moves to the next position to create the MN. In this study, the printing time was approx. 6 h and needed 42.682 voxels to produce the hollow MN with a height of 431 µm (Figure 10b).

### 5.6. Hot Embossing

The hot embossing process is a simple technique, similar to metal injection molding that include the use of heat and a PDMS mold to create a MN patch and can provide complex parts with high aspect ratios [126]. Cahill et al. fabricated 316 L stainless steel porous MNs with 36% of porosity using hot embossing of a patch of stainless steel-based feedstock. After fabrication, the MN were sintered at 1100 °C and electropolished [27]. Due to the porosity of 36% the MNs were able to wick-up 27 ± 5 μL of fluid through capillary action. The drug delivery performance of the porous 316 L stainless steel MNs (22.4 ± 4.9 mg/cm^2^) appeared to be three times higher than the topical administration (7.1 ± 4.3 mg/cm^2^). Furthermore, the metallic porous patches were able to penetrate porcine skin under a load of 19 N without leading to a failure despite the existence of porous. Porous stainless steel MNs using the hot embossing process were also developed by Ullah et al. and were coated with a thin polymer layer achieving porcine skin penetrate without fracturing, delivering calcein dye to a depth of 750 µm [127]. Comparing the coated with non-coated MNs a significant improvement in mechanical properties was observed, with the failure force reaching the 25 N. In addition to mechanical properties, the porous polymer-coated stainless steel MNs were able to store higher amount of rhodamine B and lidocaine 5 to 25-fold higher drug delivery. 

### 5.7. Metal Injection Mulding (MIM)

**Figure 11:**
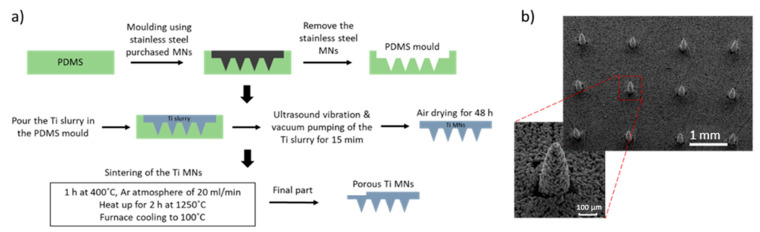
(**a**) Fabrication process of porous Ti MNs, including the fabrication of a PDMS mold, the vacuum pumping of the Ti slurry and the sintering with Argon gas continuously blowing during the sintering process. (**b**) 6 × 6 porous Ti MN arrays fabricated by the metal injection molding process with 500 μm height and 30.1% porosity [28].

The metal injection molding (MIM) process can be used as a mass-production technique for MN arrays fabrication. This technique provides the ability to manufacture parts with high precision and complex structure [128], with micro and nano featureand replication capabilities [129]. MIM is not a one-step process, since it requires numerous stages including feedstock preparation, injection molding, binder removal and the sintering (Figure 11a). The initial raw materials are metal powders combined with the binder that is injected into the mold. The metallic part is formed by the injection molding process [28]. In order to obtain parts with interconnected porosity without destroying the shape, the binder has to be removed. This process is divided into two parts, the solvent de-binding and the thermal de-binding. For the extraction of the binder, chemicals (such as heptane and hexane) can be used in the solvent de-binding process, while for the thermal process the green compact is heated up in a furnace [130]. Finally, the sintering process reduces the pore volume that has been created by the binder and that can lead to a 10–20% of shrinkage of the final part [130]. Titanium-based porous MNs (6 × 6 microneedle array with 500 μm microneedle height) were fabricated by Li et al. using MIM (Figure 11b). The titanium MNs had an average porosity of 30.1% and a pore size of 1.3 μm diameter [28]. These MNs demonstrated the ability to penetrate human forearm skin without fracturing and were able to diffuse dry rhodamine B, which was stored in the porous MNs, into rabbit skin. 

**Table 3:** Overview of the MNs fabrication techniques described in the “Fabrication Methods for Metallic MNs” section.

Fabrication Technique	Types of MNs	Material Used	Key Geometric Features	Advantages	Limitations	References
Laser Cutting	Solid, hollow	Stainless steel	Height: 700 µm, width: 200 µm Outer and inner diameter of 50 µm and 20 µm	Mass productivity, Low cost	Post-processing (i.e., electropolishing) is required (poor surface finishing)	[114,131,132]
Laser Ablation	Solid	Stainless steel Tantalum	Height >10 µm Tip diameter: 0.3–0.5 μm Aspect ratio: 1–4.5 Thickness: 2.5–10 μm	No time consuming	Required thin metallic sheet, Might cause cracks in the final structure	[118,119]
Etching	Solid Hollow	Titanium Nickel	Height: 120–250 µm Pitch: 230–280 µm	Simple process, Controllable etching rate	Chemical contamination High cost	[19,122,133]
Electroplating	Solid Hollow	Palladium Copper	Height < 500 μm Base diameter: 100–250 μm	Controlled thickness and the deposition rate	High cost	[125,134]
Hot embossing	Solid	Stainless steel Titanium	Porosity: d_90_:1.56–2.93 μm Height >300 μm Tip: 30–90 μm	Mass production, Cost effective, Complex parts	Multi-step process	[27,126]
Metal Injection molding (MIM)	Solid	Stainless steel Titanium	Porosity: d_50_: 1.3 μm Height: 460 ± 40 μm Tip diameter: 20 ± 4 μm	Mass production	Multi-step process	[28]
Direct Metal Laser Sintering (DMLS)	Solid	Stainless steel Titanium	Height: 250–700 μm Tip radius < 50 μm	Single fabrication step, Near net shaped parts, Mass production	High cost, Post-processing is required	[30,104,109,110]

## 6. Optimization of MN Structure

Several important design criteria require consideration when designing and fabricating metallic MNs. The material that is used for the MN fabrication combined with the force required during the application plays a significant role in MN insertion into the skin and in the fracture properties. The geometric characteristics of individual MNs and MN arrays have a significant effect on their mechanical and penetration properties. The most vital factors (with the exception of material type) that affect the mechanical strength are the height, diameter, thickness, aspect ratio and the shape of the MNs (summarized in Table 4) [135]. The needle height controls the depth to which a drug/vaccine may be delivered, while the aspect ratio of the needle influences ease of insertion and mechanical integrity. The tip diameter is also a key parameter influencing MN skin insertion. The penetration properties are also affected by the number of vertices of the individual MNs [136]. 

Moreover, the MN design can affect the delivery of therapeutic agents in vivo and the pain experienced by the patient during insertion. The increase of MN height can improve the drug loading, but this can be more painful during the insertion in the human body. Factors such as MN length, tip angle, thickness and width can also affect the insertion pain. Gill et al. [79] examined the effect of MN design on the pain levels experienced by human volunteers. Geometric characteristics, such as MN length, tip angle and thickness and width, were tested using different MN dimensions. The study showed that MNs with a length between 450 µm and 700 µm did not cause any pain for 100% volunteers tested and 90% of volunteers experienced pain when the MN length was increased to 1450 µm. For MNs with a length of 480 µm, changing the tip angle from 20° to 90° led to a painless penetration. Furthermore, there was no significant (*p* > 0.05) difference in pain levels reported when changing the MN width (from 160 µm to 465 µm) or the MN thickness (30 µm to 100 µm) [137]. However, the array pattern presented influenced the final characteristics in terms their application. For instance, a 5-microneedle arrays reduced the pain levels by 10% compared with hypodermic needles while 50-microneedle arrays show 10 times higher levels of pain (which corresponded to 25% of the hypodermic needle) [79]. 

Microneedle base diameter is considered as another factor that can affect both mechanical and penetration properties. The increase of the base diameter leads to higher penetration forces, which can cause the failure of the needle during insertion into the skin. According to a study that investigated the effect of the base diameter on the efficiency of the drug delivery, TTD improvement was observed when the MN base diameter was increased from 40 µm to 125 µm [138]. Consequently, increasing the base diameter led to increased mechanical stability. Conversely, keeping the base diameter constant and increasing the MN height makes them more likely to fail due to buckling. Even though a larger base diameter leads to a MN array exhibiting a higher surface for drug coating, many studies have shown that the skin permeability is not affected significantly by changing the MN geometries [82,139]. Moreover, the MN thickness is related to mechanical strength. The compression strength correlates with MN thickness, in the same way as the insertion force, since the margin of safety (i.e., ratio between MN fracture and skin insertion force) reaches the highest values with large MN thickness. Similarly, with the base diameter, the thickness does not affect the skin permeability [79].

Additionally, the needle-to-needle distance, the number of MNs within the array and the array pattern influence the penetration force [140]. Furthermore, the insertion force is also affected by MN number combined with the needle–to-needle distance, with the increase of the needle-to-needle distance leading to lower insertion forces [82,141]. The needle-to-needle distance is closely connected with the array pattern. The array pattern refers to the arrangement of the MNs within the patch, e.g., triangular, square and hexagonal MN array patterns [139]. The MN array pattern is one of the main factors that affects skin permeability. Studies have shown that the hexagonal pattern demonstrates the greatest skin permeability when compared to the square or triangular pattern arrangements. While a square pattern has a greater interface area that results in higher diffusion of the drug into the skin [142].

The recent development of porous MNs has shown potential for enhanced TDD [26]. Further optimization of these MNs is required in order to achieve a high volume of pores, while maintaining the mechanical integrity of the MN. Until recently, porous MNs have attracted less attention due to their complex fabrication processes used for their manufacture. However, recent advances in Additive Manufacturing offer great potential for manufacture of porous metallic MNs, whereby the printing parameters can be more easily adjusted to provide the desired porous properties to the final part [143].

**Table 4:** Overview of the geometrical characteristic affect the MN arrays performance.

Geometrical Characteristics	Mechanical Strength	Skin Insertion	Skin Permeability	Pain Levels	Drug Delivery	References
Needle Length	The increase of needle length (>1000 µm) can reduce compression and buckling forces.	Increasing the needle length increases the risk of bleeding during the insertion. Increasing the needle height (300–900 µm) lead to increase of penetration depth.	Increased of needle length enhances the skin permeability.	Increasing the MN length can increase the pain levels during penetration by reaching the pain receptors.	Increase of MN length can improve the drug release.	[79,137]
Needle Tip Diameter/Angle	Greater MN tip diameter increase the margin of safety (i.e., ratio between: fracture force and insertion force). Greater tip diameter increases the mechanical strength.	The small tip diameter improves the skin penetration leading to easier skin insertion.	N/A	Sharper tips lead to decrease of pain.	N/A	[19,79,82,144]
Needle Base Diameter	Increasing the base diameter (approx. >20 µm) led to increased mechanical stability.	Increasing the base diameter led to the increase of the penetration depth.	Increasing the base diameter led to effective skin permeability.	Increasing the base diameter can cause increase of pain.	Increasing the base diameter (40 µm to 125 µm) lead to improvement of TDD by increasing the drug coating.	[79,82,145]
Needle Thickness	Greater MN thickness lead to greater margin of safety. Fracture force increases with the increase of thickness.	Increasing the thickness lead to limited and more difficult skin insertion.	Increasing the thickness enhanced the effectiveness skin permeability.	Increasing the needle thickness for both solid and hollow increased the pain levels.	N/A	[19,79,82]
Aspect ratio (height: base)	High aspect ratio lead to buckling of the needles. Increasing the aspect ratio lead to decrease of mechanical strength. Stiffness increased with the decrease of aspect ratio.	Increase of aspect ratio increased the penetration depth. Low aspect ratio limited the skin penetration.	Lower aspect ratio limits the skin permeability.	N/A	Increased aspect ratio lead to increase of drug loading and release.	[82,144]
Array pattern	Increasing the needle to needle distance reduce the force during the penetration. Increasing the needle vertices (3 to 6 vertices) the needles can withstand higher compressive loads.	Increasing the needle to needle distance (30–600 μm) lead to increase of the penetration depth.	Increasing the needle vertices to 6 increases the skin permeability.	Increasing the number of MNs in the array lead to increase of pain.	N/A	[79,82,137,146]

## 7. Summary and Future Perspectives

Considering the need and the disadvantages of the existing TDD systems for the treatment and diagnosis of different diseases and incidence, a micro sized device presents significant potential as a painless minimal invasive device. Several TDD systems have been developed (e.g., conventional hypodermic needles, oral administration, topical creams and non-invasive transdermal patches) to date, to overcome these drawbacks. However, these systems still lack the ability to overcome the main challenge, which is painless penetration without the fracture of the device and at the same time, controlled and continuous or rapid drug release. MN arrays have the potential to offer advantageous properties including good bioavailability, enhanced skin permeability [13] without skin irritation or allergic reactions, while at the same time combining the requirement for painless application and controlled drug delivery. 

To date, a range of such devices have been investigated for use in the treatment of several diseases (e.g., treatment of diabetes, cancer diagnosis and therapy, chronic pain treatment and control/treatment of obesity). However, these studies have been mainly focused on polymeric and dissolvable needles. For instance, studies reported the use of polymeric MNs for the diagnosis and therapy of Type 1 and Type 2 diabetes [34,36] and for the treatment of multiple tumors as anticancer therapeutic approaches [13]. Although, these polymeric MNs offer a significant advantage as they can function as painless TDD systems, they have shown difficulties in penetrating the skin, insufficient biocompatibility and poor drug loading capacity. Currently, the scientific knowledge and understanding of the fabrication method required and the design rules associated with mechanical properties and geometrical characteristics is limited and considerable research efforts are being invested. 

Initially, investigation demonstrated that the development of polymer-based MNs obtain poor mechanical properties with forces that lead to tip fracture before skin penetration. Metallic MNs show potential to overcome these challenges and are able to deliver relatively larger amounts of drugs (e.g., via drug coating or the drug loading of porous MNs) with higher mechanical integrity and piercing strength. In particular, recent studies have shown that the manufacture of solid metallic-based MNs can achieve optimal geometric characteristics for constant and controlled drug delivery [147]. However, since MNs can be manufactured in a number of shapes and types, MNs with optimal geometric characteristics can combine increased mechanical strength and reduced penetration forces in order to penetrate the stratum corneum, one of the main barriers of the skin. Nevertheless, there are challenges remaining in this area so, further investigations into the ideal properties, such as porosity and density, of the solid metallic-based MN arrays are required. For instance, there is a need for new research in order to achieve optimal mechanical properties as well as drug loading ability and biological performance, and ultimately improved disease treatment and management.

Additive Manufacturing is a subversive, innovative technology and fast-growing field that has introduced new opportunities in the area of personalized medication, offering the potential for high impact in terms of the efficiency, quality and cost-effectiveness in the production of pharmaceutical therapies and biomaterial-based devices [104,148]. However, many limitations related to the final geometrical and physical properties (e.g., poor surface finishing, porosity and shape) and the process (e.g., multi-step process, high cost and risk of chemical contamination) should be addressed to employ Additive Manufacturing as a successful approach for TDD. 

Research development of the metallic MN arrays is focused on exploring its potential as a drug delivery system for the controlled and localized delivery of cells, growth factors and small molecules focusing on the synchronization of the load and the release of these elements. Another area of research focus relates to the investigation and optimization of the additive manufacturing process in order to achieve improved penetration and mechanical properties. Considerable research remains on improving the suboptimal mechanical, physical properties (e.g., compression strength, bulk modulus and ultimate strain before failure) and drug release rate and/or amount. Therefore, future studies should focus on the understanding of the fundamental principles of AM, such as the enhancement of laser beam or nozzle features, leading to the ultimate resolution of printed MNs [149]. This new knowledge will be very important to ensure the ease of handling, safety and effectiveness of the product(s) developed leading to the successful fabrication of MN arrays and understanding the structure-property-performance relationship.

Consequently, the development of MNs as drug delivery systems using upgraded Additive Manufacturing systems is necessary to promote their quality. The development of MNs through 3D printing can enhance accessibility offering customization and cost-efficiency using low-cost raw printing materials. Furthermore, the ability to control the printing resolution can improve the accuracy of the final features. Therefore, metallic 3D printed MN arrays have gained increasing significance in recent years since they demonstrate potential as TDD systems for painless application and controlled, continuous or rapid drug release for a number of challenging diseases [150]. Furthermore, scientific findings should be combined with commercialization schemes, in order to ensure rapid bench-to-bedside translation is achieved and that patients can quickly benefit from these new promising technologies.

## Figures and Tables

**Table 2:** Mechanical properties of metals used for the manufacture of metal-based MN arrays [96].

Metals	Young’s Modulus (GPa)	Ultimate Tensile Strength (MPa)	Elongation (%)
Nickel	207	45–450	30–47
Palladium	117	180–320	30–40
Platinum	171	125–165	35
Tantalum	175–190	760	30
Copper	130	193–262	30
Pure Titanium	102–120	240–550	15–30
Ti6Al4V	114	1170	10
Stainless steel	193–200	505–1000	60–70

## Data Availability

Not applicable.

## References

[1] Waghule T., Singhvi G., Dubey S.K., Pandey M.M., Gupta G., Singh M., Dua K. (2019). Microneedles: A smart approach and increasing potential for transdermal drug delivery system. Biomed. Pharmacother..

[2] Viswanathan P., Muralidaran Y., Ragavan G. (2017). Challenges in oral drug delivery: A nano-based strategy to overcome. Nanostructures for Oral Medicine.

[3] Cross S., Roberts M. (2005). Physical Enhancement of Transdermal Drug Application: Is Delivery Technology Keeping up with Pharmaceutical Development?. Curr. Drug Deliv..

[4] Kaur L.P., Guleri T.K. (2013). Topical Gel: A Recent Approach for Novel Drug Delivery. Asian J. Biomed. Pharm. Sci..

[5] Patil P., Datir S., Saudagar R. (2019). A Review on Topical Gels as Drug Delivery System. J. Drug Deliv. Ther..

[6] Chander Jhawat V., Saini V., Kamboj S., Maggon N. (2013). Transdermal drug delivery systems: Approaches and advancements in drug absorption through skin. Int. J. Pharm. Sci. Rev. Res..

[7] Chhatrani B.M., Shah D.P., Lalbhai N. (2017). Naranjibhai A Review on Microemulsion Based Gel: A Novel Approach for Enhancing Topical Delivery of Hydrophobic Drug. Int. J. Pharm. Pharm. Res..

[8] Patel D., Chaudhary S.A., Parmar B., Bhura N. (2012). Transdermal Drug Delivery System: A Review. Pharma Innov..

[9] Donnelly R.F., Singh T.R.R., Garland M.J., Migalska K., Majithiya R., McCrudden C.M., Kole P.L., Mahmood T.M.T., McCarthy H.O., Woolfson A.D. (2012). Hydrogel-forming microneedle arrays for enhanced transdermal drug delivery. Adv. Funct. Mater..

[10] Arunachalam A., Karthikeyan M., Kumar D. (2010). Review Article Current Pharma Research Transdermal Drug Delivery System: A Review. J. Curr. Pharma Res..

[11] Shingade G.M. (2012). Review on: Recent Trend on Transdermal Drug Delivery System. J. Drug Deliv. Ther..

[12] Khanna P., Strom J.A., Malone J.I., Bhansali S. (2008). Microneedle-based automated therapy for diabetes mellitus. J. Diabetes Sci. Technol..

[13] Moreira A.F., Rodrigues C.F., Jacinto T.A., Miguel S.P., Costa E.C., Correia I.J. (2019). Microneedle-based delivery devices for cancer therapy: A review. Pharmacol. Res..

[14] Hao Y., Li W., Zhou X.L., Yang F., Qian Z.Y. (2017). Microneedles-based transdermal drug delivery systems: A review. J. Biomed. Nanotechnol..

[15] Makvandi P., Kirkby M., Hutton A.R.J., Shabani M., Yiu C.K.Y., Baghbantaraghdari Z., Jamaledin R., Carlotti M., Mazzolai B., Mattoli V. (2021). Engineering Microneedle Patches for Improved Penetration: Analysis, Skin Models and Factors Affecting Needle Insertion.

[16] Chen M.C., Ling M.H., Kusuma S.J. (2015). Poly-γ-glutamic acid microneedles with a supporting structure design as a potential tool for transdermal delivery of insulin. Acta Biomater..

[17] Thomas D. (2016). Costs, Benefits, and Adoption of Additive Manufacturing: A Supply Chain Perspective. Int. J. Adv. Manuf. Technol..

[18] Donnelly R.F., Singh T.R.R., Morrow D.I.J., Woolfson A.D. (2012). Microneedles: Design, Microfabrication and Optimization. Microneedle-Mediated Transdermal and Intradermal Drug Delivery.

[19] Donnelly R.F., Raj Singh T.R., Woolfson A.D. (2010). Microneedle-based drug delivery systems: Microfabrication, drug delivery, and safety. Drug Deliv..

[20] Tuan-Mahmood T.M., McCrudden M.T.C., Torrisi B.M., McAlister E., Garland M.J., Singh T.R.R., Donnelly R.F. (2013). Microneedles for intradermal and transdermal drug delivery. Eur. J. Pharm. Sci..

[21] Dharadhar S., Majumdar A., Dhoble S. (2019). Microneedles for transdermal drug delivery: A systematic review. Drug Dev. Ind. Pharm..

[22] He X., Sun J., Zhuang J., Xu H., Liu Y., Wu D. (2019). Microneedle System for Transdermal Drug and Vaccine Delivery: Devices, Safety, and Prospects. Dose-Response.

[23] Yang J., Liu X., Fu Y., Song Y. (2019). Recent advances of microneedles for biomedical applications: Drug delivery and beyond. Acta Pharm. Sin. B.

[24] Zhou C.P., Liu Y.L., Wang H.L., Zhang P.X., Zhang J.L. (2010). Transdermal delivery of insulin using microneedle rollers in vivo. Int. J. Pharm..

[25] Matriano J.A., Cormier M., Johnson J., Young W.A., Buttery M., Nyam K., Daddona P.E. (2002). Macroflux^®^ microprojection array patch technology: A new and efficient approach for intracutaneous immunization. Pharm. Res..

[26] Bao L., Park J., Bonfante G., Kim B. (2022). Recent advances in porous microneedles: Materials, fabrication, and transdermal applications. Drug Deliv. Transl. Res..

[27] Cahill E.M., Keaveney S., Stuettgen V., Eberts P., Ramos-Luna P., Zhang N., Dangol M., O’Cearbhaill E.D. (2018). Metallic microneedles with interconnected porosity: A scalable platform for biosensing and drug delivery. Acta Biomater..

[28] Li J., Liu B., Zhou Y., Chen Z., Jiang L., Yuan W., Liang L. (2017). Fabrication of a Ti porous microneedle array by metal injection molding for transdermal drug delivery. PLoS ONE.

[29] van der Maaden K., Luttge R., Vos P.J., Bouwstra J., Kersten G., Ploemen I. (2015). Microneedle-based drug and vaccine delivery via nanoporous microneedle arrays. Drug Deliv. Transl. Res..

[30] Aldawood F.K., Andar A., Desai S. (2021). A comprehensive review of microneedles: Types, materials, processes, characterizations and applications. Polymers.

[31] WHO Regional Office for Europe (2017). WHO European Regional Diabetes Report: Diabetes Mellitus—Epidemiology, Prevention and Control.

[32] American Diabetes Association (2010). Diagnosis and classification of diabetes mellitus. Diabetes Care.

[33] Kong J.E., Koh J., Lin J., Di Carlo D. (2015). Research highlights: Translating chips. Lab A Chip.

[34] Beirne P.V., Hennessy S., Cadogan S.L., Shiely F., Fitzgerald T., Macleod F. (2018). Needle size for vaccination procedures in children and adolescents. Cochrane Database Syst. Rev..

[35] Paudel K.S., Milewski M., Swadley C.L., Brogden N.K., Ghosh P.S.A. (2010). Challenges and opportunities in dermal/transdermal delivery. Ther Deliv..

[36] Chen W., Wang G., Yung B.C., Liu G., Qian Z., Chen X. (2017). Long-Acting Release Formulation of Exendin-4 Based on Biomimetic Mineralization for Type 2 Diabetes Therapy. ACS Nano.

[37] Yu J., Zhang Y., Ye Y., DiSanto R., Sun W., Ranson D., Ligler F.S., Buse J.B., Gu Z., Ho D. (2015). Microneedle-array patches loaded with hypoxia-sensitive vesicles provide fast glucose-responsive insulin delivery. Proc. Natl. Acad. Sci. USA.

[38] Steil G.M., Panteleon A.E., Rebrin K. (2004). Closed-loop insulin delivery—The path to physiological glucose control. Adv. Drug Deliv. Rev..

[39] Renard E. (2002). Implantable closed-loop glucose-sensing and insulin delivery: The future for insulin pump therapy. Curr. Opin. Pharmacol..

[40] Liu S., Wu D., Quan Y.S., Kamiyama F., Kusamori K., Katsumi H., Sakane T., Yamamoto A. (2016). Improvement of Transdermal Delivery of Exendin-4 Using Novel Tip-Loaded Microneedle Arrays Fabricated from Hyaluronic Acid. Mol. Pharm..

[41] Lee H., Choi T.K., Lee Y.B., Cho H.R., Ghaffari R., Wang L., Choi H.J., Chung T.D., Lu N., Hyeon T. (2016). A graphene-based electrochemical device with thermoresponsive microneedles for diabetes monitoring and therapy. Nat. Nanotechnol..

[42] Ling M.H., Chen M.C. (2013). Dissolving polymer microneedle patches for rapid and efficient transdermal delivery of insulin to diabetic rats. Acta Biomater..

[43] Invernale M.A., Tang B.C., York R.L., Le L., Hou D.Y., Anderson D.G. (2014). Microneedle Electrodes Toward an Amperometric Glucose-Sensing Smart Patch. Adv. Healthc. Mater..

[44] Davis S.P., Martanto W., Allen M.G., Prausnitz M.R. (2005). Hollow metal microneedles for insulin delivery to diabetic rats. IEEE Trans. Biomed. Eng..

[45] Martanto W., Davis S.P., Holiday N.R., Wang J., Gill H.S., Prausnitz M.R. (2004). Transdermal delivery of insulin using microneedles in vivo. Pharm. Res..

[46] Yan X.X., Liu J.Q., Jiang S.D., Yang B., Yang C.S. (2013). Fabrication and testing of porous Ti microneedles for drug delivery. Micro Nano Lett..

[47] Zhang Y., Yu J., Kahkoska A.R., Wang J., Buse J.B., Gu Z. (2019). Advances in transdermal insulin delivery. Adv. Drug Deliv. Rev..

[48] Demir Y.K., Akan Z., Kerimoglu O. (2013). Characterization of Polymeric Microneedle Arrays for Transdermal Drug Delivery. PLoS ONE.

[49] WHO (2022). WHO European Regional Obesity Report 2022.

[50] Zhang Y., Yu J., Wen D., Chen G., Gu Z. (2018). The potential of a microneedle patch for reducing obesity. Expert Opin. Drug Deliv..

[51] Malik V.S., Willett W.C., Hu F.B. (2013). Global obesity: Trends, risk factors and policy implications. Nat. Rev. Endocrinol..

[52] Friedman J.M. (2009). Obesity: Causes and control of excess body fat. Nature.

[53] Melnikova I., Wages D. (2006). Anti-obesity therapies. Nat. Rev. Drug Discov..

[54] Dangol M., Kim S., Li C.G., Fakhraei Lahiji S., Jang M., Ma Y., Huh I., Jung H. (2017). Anti-obesity effect of a novel caffeine-loaded dissolving microneedle patch in high-fat diet-induced obese C57BL/6J mice. J. Control. Release.

[55] Hiradate R., Khalil I.A., Matsuda A., Sasaki M., Hida K., Harashima H. (2021). A novel dual-targeted rosiglitazone-loaded nanoparticle for the prevention of diet-induced obesity via the browning of white adipose tissue. J. Control. Release.

[56] Zhang Y., Liu Q., Yu J., Yu S., Wang J., Qiang L., Gu Z. (2017). Locally Induced Adipose Tissue Browning by Microneedle Patch for Obesity Treatment. ACS Nano.

[57] An S.M., Seong K.Y., Yim S.G., Hwang Y.J., Bae S.H., Yang S.Y., An B.S. (2018). Intracutaneous delivery of gelatins induces lipolysis and suppresses lipogenesis of adipocytes. Acta Biomater..

[58] Bray F., Ferlay J., Soerjomataram I., Siegel R.L., Torre L.A., Jemal A. (2018). Global cancer statistics 2018: GLOBOCAN estimates of incidence and mortality worldwide for 36 cancers in 185 countries. CA. Cancer J. Clin..

[59] Chen M.C., Lin Z.W., Ling M.H. (2016). Near-infrared light-activatable microneedle system for treating superficial tumors by combination of chemotherapy and photothermal therapy. ACS Nano.

[60] Dong L., Li Y., Li Z., Xu N., Liu P., Du H., Zhang Y., Huang Y., Zhu J., Ren G. (2018). Au Nanocage-Strengthened Dissolving Microneedles for Chemo-Photothermal Combined Therapy of Superficial Skin Tumors. ACS Appl. Mater. Interfaces.

[61] Ye Y., Wang J., Hu Q., Hochu G.M., Xin H., Wang C., Gu Z. (2016). Synergistic Transcutaneous Immunotherapy Enhances Antitumor Immune Responses through Delivery of Checkpoint Inhibitors. ACS Nano.

[62] van der Maaden K., Heuts J., Camps M., Pontier M., Terwisscha van Scheltinga A., Jiskoot W., Ossendorp F., Bouwstra J. (2018). Hollow microneedle-mediated micro-injections of a liposomal HPV E743–63 synthetic long peptide vaccine for efficient induction of cytotoxic and T-helper responses. J. Control. Release.

[63] Singh V., Kesharwani P. (2021). Recent advances in microneedles-based drug delivery device in the diagnosis and treatment of cancer. J. Control. Release.

[64] Chablani L., Tawde S.A., Akalkotkar A., D’Souza M.J. (2019). Evaluation of a Particulate Breast Cancer Vaccine Delivered via Skin. AAPS J..

[65] Tawde S.A., Chablani L., Akalkotkar A., D’Souza M.J. (2016). Evaluation of microparticulate ovarian cancer vaccine via transdermal route of delivery. J. Control. Release.

[66] Yan G., Warner K.S., Zhang J., Sharma S., Gale B.K. (2010). Evaluation needle length and density of microneedle arrays in the pretreatment of skin for transdermal drug delivery. Int. J. Pharm..

[67] Gill H.S., Prausnitz M.R. (2007). Coated microneedles for transdermal delivery. J. Control. Release.

[68] Xie X., Pascual C., Lieu C., Oh S., Wang J., Zou B., Xie J., Li Z., Xie J., Yeomans D.C. (2017). Analgesic Microneedle Patch for Neuropathic Pain Therapy. ACS Nano.

[69] Mills S.E.E., Nicolson K.P., Smith B.H. (2019). Chronic pain: A review of its epidemiology and associated factors in population-based studies. Br. J. Anaesth..

[70] Kochhar J.S., Lim W.X.S., Zou S., Foo W.Y., Pan J., Kang L. (2013). Microneedle integrated transdermal patch for fast onset and sustained delivery of lidocaine. Mol. Pharm..

[71] Chen M.C., Chan H.A., Ling M.H., Su L.C. (2017). Implantable polymeric microneedles with phototriggerable properties as a patient-controlled transdermal analgesia system. J. Mater. Chem. B.

[72] Pan J., Ruan W., Qin M., Long Y., Wan T., Yu K., Zhai Y., Wu C., Xu Y. (2018). Intradermal delivery of STAT3 siRNA to treat melanoma via dissolving microneedles. Sci. Rep..

[73] Cole G., Ali A.A., McCrudden C.M., McBride J.W., McCaffrey J., Robson T., Kett V.L., Dunne N.J., Donnelly R.F., McCarthy H.O. (2018). DNA vaccination for cervical cancer: Strategic optimisation of RALA mediated gene delivery from a biodegradable microneedle system. Eur. J. Pharm. Biopharm..

[74] Kim N.W., Kim S.Y., Lee J.E., Yin Y., Lee J.H., Lim S.Y., Kim E.S., Duong H.T.T., Kim H.K., Kim S. (2018). Enhanced Cancer Vaccination by in Situ Nanomicelle-Generating Dissolving Microneedles. ACS Nano.

[75] Ali A.A., Mccrudden C.M., Mccaffrey J., Mcbride J.W., Cole G., Dunne N.J., Robson T., Kissenpfennig A., Donnelly R.F., Mccarthy H.O. (2016). DNA Vaccination for Cervical Cancer; A Novel Technology Platform of NU. Nanomed. Nanotechnol. Biol. Med..

[76] Wang C., Ye Y., Hochu G.M., Sadeghifar H., Gu Z. (2016). Enhanced Cancer Immunotherapy by Microneedle Patch-Assisted Delivery of Anti-PD1 Antibody. Nano Lett..

[77] Zaric M., Lyubomska O., Touzelet O., Poux C., Al-Zahrani S., Fay F., Wallace L., Terhorst D., Malissen B., Henri S. (2013). Skin dendritic cell targeting via microneedle arrays laden with antigen-encapsulated poly-D, l-Lactide-Co-Glycolide nanoparticles induces efficient antitumor and antiviral immune responses. ACS Nano.

[78] Demuth P.C., Su X., Samuel R.E., Hammond P.T., Irvine D.J. (2010). Nano-layered microneedles for transcutaneous delivery of polymer nanoparticles and plasmid DNA. Adv. Mater..

[79] Gill H.S., Denson D.D., Burris B.A., Prausnitz M.R. (2008). Effect of microneedle design on pain in human volunteers. Clin. J. Pain.

[80] Yeu-Chun K., Fu-Shi Q., Richard C., Sang-Moo K., Mark P. (2014). Formulation and coating of microneedles with inactivated influenza virus to improve vaccine stability and immunogenicity. Bone.

[81] Conci A., Brazil A.L., Popovici D., Jiga G., Lebon F. (2018). Modeling the behavior of human body tissues on penetration. AIP Conf. Proc..

[82] Davidson A., Al-Qallaf B., Das D.B. (2008). Transdermal drug delivery by coated microneedles: Geometry effects on effective skin thickness and drug permeability. Chem. Eng. Res. Des..

[83] Kawanaka K., Uetsuji Y., Tsuchiya K., Nakamachi E. (2008). Development of automatic blood extraction device with a micro-needle for blood-sugar level measurement. Smart Struct. Devices Syst. IV.

[84] Li T., Barnett A., Rogers K.L., Gianchandani Y.B. (2009). A blood sampling microsystem for pharmacokinetic applications: Design, fabrication, and initial results. Lab A Chip.

[85] Liu G.S., Kong Y., Wang Y., Luo Y., Fan X., Xie X., Yang B.R., Wu M.X. (2020). Microneedles for transdermal diagnostics: Recent advances and new horizons. Biomaterials.

[86] Mishra R., Maiti T.K., Bhattacharyya T.K. (2018). Design and Scalable Fabrication of Hollow SU-8 Microneedles for Transdermal Drug Delivery. IEEE Sens. J..

[87] Kim K., Lee J.B. (2007). High aspect ratio tapered hollow metallic microneedle arrays with microfluidic interconnector. Microsyst. Technol..

[88] Madden J., O’Mahony C., Thompson M., O’Riordan A., Galvin P. (2020). Biosensing in dermal interstitial fluid using microneedle based electrochemical devices. Sens. Bio-Sens. Res..

[89] Cahill E.M. (2019). Porous Metallic Microneedles for Drug Delivery and Bio-Sensing. Ph.D. Thesis.

[90] Aksit A., Arteaga D.N., Arriaga M., Wang X., Watanabe H., Kasza K.E., Lalwani A.K., Kysar J.W. (2018). In-vitro perforation of the round window membrane via direct 3-D printed microneedles. Biomed. Microdevices.

[91] Park J.H., Allen M.G., Prausnitz M.R. (2005). Biodegradable polymer microneedles: Fabrication, mechanics and transdermal drug delivery. J. Control. Release.

[92] Sadeqi A., Kiaee G., Zeng W., Rezaei Nejad H., Sonkusale S. (2022). Hard polymeric porous microneedles on stretchable substrate for transdermal drug delivery. Sci. Rep..

[93] Aksit A., Rastogi S., Nadal M.L., Parker A.M., Lalwani A.K., West A.C., Kysar J.W. (2020). Drug delivery device for the inner ear: Ultra-sharp fully metallic microneedles. Drug Deliv. Transl. Res..

[94] Jiang Q., Reddy N., Yang Y. (2010). Cytocompatible cross-linking of electrospun zein fibers for the development of water-stable tissue engineering scaffolds. Acta Biomater..

[95] Donnelly R.F., Singh T.R.R., Morrow D.I.J., Woolfson A.D. (2015). Microneedle applications in improving skin appearance. Exp. Dermatol..

[96] Ryan F.D. (2018). Microneedles for Drug and Vaccine Delivery and Patient Monitoring.

[97] Dou X., Liu L.-L., Zhu X.-J. (2003). Nickel-elicited systemic contact dermatitis. Contact Dermat..

[98] Raison-Peyron N., Guillard O., Khalil Z., Guilhou J.J., Guillot B. (2005). Nickel-elicited systemic contact dermatitis from a peripheral intravenous catheter. Contact Dermat..

[99] Gawkrodger D. (1996). Nickel dermatitis: How much nickel is safe?. Contact Dermat..

[100] Verbaan F.J., Bal S.M., van den Berg D.J., Groenink W.H.H., Verpoorten H., Lüttge R., Bouwstra J.A. (2007). Assembled microneedle arrays enhance the transport of compounds varying over a large range of molecular weight across human dermatomed skin. J. Control. Release.

[101] Gad S.C., McCord M.G. (2008). Safety Evaluation in the Development of Medical Devices and Combination Products.

[102] Larrañeta E., Lutton R.E.M., Woolfson A.D., Donnelly R.F. (2016). Microneedle arrays as transdermal and intradermal drug delivery systems: Materials science, manufacture and commercial development. Mater. Sci. Eng. R Rep..

[103] Niinomi M., Nakai M. (2011). Titanium-based biomaterials for preventing stress shielding between implant devices and bone. Int. J. Biomater..

[104] Krieger K.J., Liegey J., Cahill E.M., Bertollo N., Lowery M.M., O’Cearbhaill E.D. (2020). Development and Evaluation of 3D-Printed Dry Microneedle Electrodes for Surface Electromyography. Adv. Mater. Technol..

[105] Wermeling D.P., Banks S.L., Hudson D.A., Gill H.S., Gupta J., Prausnitz M.R., Stinchcomb A.L. (2008). Microneedles permit transdermal delivery of a skin-impermeant medication to humans. Proc. Natl. Acad. Sci. USA.

[106] Parker E.R., Rao M.P., Turner K.L., MacDonald N.C. Bulk titanium microneedles with embedded microfluidic networks for transdermal drug delivery. Proceedings of the IEEE International Conference on Micro Electro Mechanical Systems (MEMS).

[107] Parker E.R., Rao M.P., Turner K.L., Meinhart C.D., MacDonald N.C. (2007). Bulk micromachined titanium microneedles. J. Microelectromechanical Syst..

[108] Chen Q., Thouas G. (2014). Biomaterials: A Basic Introduction.

[109] Atzeni E., Salmi A. (2015). Study on unsupported overhangs of AlSi10Mg parts processed by Direct Metal Laser Sintering (DMLS). J. Manuf. Process..

[110] Keshavarzkermani A., Sadowski M., Ladani L. (2018). Direct metal laser melting of Inconel 718: Process impact on grain formation and orientation. J. Alloys Compd..

[111] Jardini A.L., Larosa M.A., Filho R.M., Zavaglia C.A.D.C., Bernardes L.F., Lambert C.S., Calderoni D.R., Kharmandayan P. (2014). Cranial reconstruction: 3D biomodel and custom-built implant created using additive manufacturing. J. Cranio-Maxillofac. Surg..

[112] Laverty D.P., Thomas M.B.M., Clark P., Addy L.D. (2016). The use of 3D metal printing (direct metal laser sintering) in removable prosthodontics. Dent. Update.

[113] Panda B.K., Sahoo S. (2019). Thermo-mechanical modeling and validation of stress field during laser powder bed fusion of AlSi10Mg built part. Results Phys..

[114] Uddin M.J., Scoutaris N., Klepetsanis P., Chowdhry B., Prausnitz M.R., Douroumis D. (2015). Inkjet printing of transdermal microneedles for the delivery of anticancer agents. Int. J. Pharm..

[115] Pere C.P.P., Economidou S.N., Lall G., Ziraud C., Boateng J.S., Alexander B.D., Lamprou D.A., Douroumis D. (2018). 3D printed microneedles for insulin skin delivery. Int. J. Pharm..

[116] Choi S.O., Kim Y.C., Park J.H., Hutcheson J., Gill H.S., Yoon Y.K., Prausnitz M.R., Allen M.G. (2010). An electrically active microneedle array for electroporation. Biomed. Microdevices.

[117] Bhattacharya S., Kam D.H., Song L., Mazumder J. (2012). Characterization of individual microneedles formed on alloy surfaces by femtosecond laser ablation. Metall. Mater. Trans. A Phys. Metall. Mater. Sci..

[118] Omatsu T., Chujo K., Miyamoto K., Okida M., Nakamura K., Aoki N., Morita R. (2010). Metal microneedle fabrication using twisted light with spin. Opt. Express.

[119] Omatsu T., Miyamoto K., Morita R. (2017). Optical Vortices Illumination Enables the Creation of Chiral Nanostructures. Vortex Dynamics and Optical Vortices.

[120] Cannon B., Nedergaard J. (2004). Brown Adipose Tissue: Function and Physiological Significance. Physiol. Rev..

[121] Dragicevic N., Maibach H.I. (2017). Percutaneous Penetration Enhancers Physical Methods in Penetration Enhancement.

[122] Shikida M., Hasada T., Sato K. (2006). Fabrication of a hollow needle structure by dicing, wet etching and metal deposition. J. Micromech. Microeng..

[123] Mann R.P. (2012). Encyclopedia of Nanotechnology.

[124] Wilke N., Mulcahy A., Ye S.R., Morrissey A. (2005). Process optimization and characterization of silicon microneedles fabricated by wet etch technology. Microelectron. J..

[125] Sachan R., Schürch P., Testa P., Hepp E., Koelmans W.W., Narayan R.J. (2021). Hollow copper microneedle made by local electrodeposition-based additive manufacturing. MRS Adv..

[126] Juster H., van der Aar B., de Brouwer H. (2019). A review on microfabrication of thermoplastic polymer-based microneedle arrays. Polym. Eng. Sci..

[127] Ullah A., Kim C.M., Kim G.M. (2018). Porous polymer coatings on metal microneedles for enhanced drug delivery. R. Soc. Open Sci..

[128] Whiteside B.R., Martyn M.T., Coates P.D., Allan P.S., Hornsby P.R., Greenway G. (2003). Micromoulding: Process characteristics and product properties. Plast. Rubber Compos..

[129] Nair K., Whiteside B., Grant C., Patel R., Tuinea-Bobe C., Norris K., Paradkar A. (2015). Investigation of plasma treatment on micro-injection moulded microneedle for drug delivery. Pharmaceutics.

[130] Hamidi M.F.F.A., Harun W.S.W., Samykano M., Ghani S.A.C., Ghazalli Z., Ahmad F., Sulong A.B. (2017). A review of biocompatible metal injection moulding process parameters for biomedical applications. Mater. Sci. Eng. C.

[131] Hara Y., Yamada M., Tatsukawa C., Takahashi T., Suzuki M., Aoyagi S. (2016). Fabrication of stainless steel microneedle with laser-cut sharp tip and its penetration and blood sampling performance. Int. J. Autom. Technol..

[132] Eltawahni H.A., Hagino M., Benyounis K.Y., Inoue T., Olabi A.G. (2012). Effect of CO 2 laser cutting process parameters on edge quality and operating cost of AISI316L. Opt. Laser Technol..

[133] Zhang S., Zeng X., Matthews D.T.A., Igartua A., Rodriguez-Vidal E., Contreras Fortes J., Saenz de Viteri V., Pagano F., Wadman B., Wiklund E.D. (2016). Selection of micro-fabrication techniques on stainless steel sheet for skin friction. Friction.

[134] Azmi A.A., Jai J., Zamanhuri N.A., Yahya A. (2018). Precious Metals Recovery from Electroplating Wastewater: A Review. IOP Conf. Ser. Mater. Sci. Eng..

[135] Karatutlu A., Barhoum A., Sapelkin A. (2018). Liquid-Phase Synthesis of Nanoparticles and Nanostructured Materials.

[136] Norman J.J., Choi S.O., Tong N.T., Aiyar A.R., Patel S.R., Prausnitz M.R., Allen M.G. (2013). Hollow microneedles for intradermal injection fabricated by sacrificial micromolding and selective electrodeposition. Biomed. Microdevices.

[137] Donnelly R.F., Garland M.J., Morrow D.I.J., Migalska K., Singh T.R.R., Majithiya R., Woolfson A.D. (2010). Optical coherence tomography is a valuable tool in the study of the effects of microneedle geometry on skin penetration characteristics and in-skin dissolution. J. Control. Release.

[138] Badran M.M., Kuntsche J., Fahr A. (2009). Skin penetration enhancement by a microneedle device (Dermaroller®) in vitro: Dependency on needle size and applied formulation. Eur. J. Pharm. Sci..

[139] Basile A., Gallucci F. (2009). Membrane reactors—Part I. Asia-Pacific J. Chem. Eng..

[140] Chandrasekaran S., Frazier A.B. (2003). Characterization of surface micromachined metallic microneedles. J. Microelectromechanical Syst..

[141] Park J.H., Yoon Y.K., Choi S.O., Prausnitz M.R., Allen M.G. (2007). Tapered conical polymer microneedles fabricated using an integrated lens technique for transdermal drug delivery. IEEE Trans. Biomed. Eng..

[142] Johnson A.R., Caudill C.L., Tumbleston J.R., Bloomquist C.J., Moga K.A., Ermoshkin A., Shirvanyants D., Mecham S.J., Luft J.C., De Simone J.M. (2016). Single-step fabrication of computationally designed microneedles by continuous liquid interface production. PLoS ONE.

[143] Jeon T.J., Hwang T.W., Yun H.J., VanTyne C.J., Moon Y.H. (2018). Control of porosity in parts produced by a direct laser melting process. Appl. Sci..

[144] Gittard S.D., Chen B., Xu H., Ovsianikov A., Chichkov B.N., Monteiro-Riviere N.A., Narayan R.J. (2013). The effects of geometry on skin penetration and failure of polymer microneedles. J. Adhes. Sci. Technol..

[145] Kochhar J.S., Quek T.C., Soon W.J., Choi J., Zou S., Kang L. (2013). Effect of microneedle geometry and supporting substrate on microneedle array penetration into skin. J. Pharm. Sci..

[146] Loizidou E.Z., Inoue N.T., Ashton-Barnett J., Barrow D.A., Allender C.J. (2016). Evaluation of geometrical effects of microneedles on skin penetration by CT scan and finite element analysis. Eur. J. Pharm. Biopharm..

[147] Jung J.H., Jin S.G. (2021). Microneedle for transdermal drug delivery: Current trends and fabrication. J. Pharm. Investig..

[148] Shu W., Heimark H., Bertollo N., Tobin D.J., O’Cearbhaill E.D., Annaidh A.N. (2021). Insights into the mechanics of solid conical microneedle array insertion into skin using the finite element method. Acta Biomater..

[149] Krieger K.J., Bertollo N., Dangol M., Sheridan J.T., Lowery M.M., O’Cearbhaill E.D. (2019). Simple and customizable method for fabrication of high-aspect ratio microneedle molds using low-cost 3D printing. Microsystems Nanoeng..

[150] Dabbagh S.R., Sarabi M.R., Rahbarghazi R., Sokullu E., Yetisen A.K., Tasoglu S. (2021). 3D-printed microneedles in biomedical applications. iScience.

